# Identification and validation of eight lysosomes-related genes signatures and correlation with immune cell infiltration in lung adenocarcinoma

**DOI:** 10.1186/s12935-023-03149-5

**Published:** 2023-12-13

**Authors:** Dingli Song, Lili Zhao, Guang Zhao, Qian Hao, Jie Wu, Hong Ren, Boxiang Zhang

**Affiliations:** 1 Department of Thoracic Surgery, The First Affiliated Hospital of Xi’an Jiaotong University, Xi’an, China; 2 Department of Neurology, The Second Affiliated Hospital of Xi’an Jiaotong University, Xi’an, China; 3 Department of Oncology, The Second Affiliated Hospital of Xi’an Jiaotong University, Xi’an, China

**Keywords:** Lysosomes, Lung adenocarcinoma, Immune infiltration, Signature

## Abstract

Lung cancer is the leading cause of cancer-related death. Lysosomes are key degradative compartments that maintain protein homeostasis. In current study, we aimed to construct a lysosomes-related genes signature to predict the overall survival (OS) of patients with Lung Adenocarcinoma (LUAD). Differentially expressed lysosomes-related genes (DELYs) were analyzed using The Cancer Genome Atlas (TCGA-LUAD cohort) database. The prognostic risk signature was identified by Least Absolute Shrinkage and Selection Operator (LASSO)-penalized Cox proportional hazards regression and multivariate Cox analysis. The predictive performance of the signature was assessed by Kaplan–Meier curves and Time-dependent receiver operating characteristic (ROC) curves. Gene set variant analysis (GSVA) was performed to explore the potential molecular biological function and signaling pathways. ESTIMATE and single sample gene set enrichment analysis (ssGSEA) were applied to estimate the difference of tumor microenvironment (TME) between the different risk subtypes. An eight prognostic genes (ACAP3, ATP8B3, BTK, CAV2, CDK5R1, GRIA1, PCSK9, and PLA2G3) signature was identified and divided patients into high-risk and low-risk groups. The prognostic signature was an independent prognostic factor for OS (HR > 1, *p* < 0.001). The molecular function analysis suggested that the signature was significantly correlated with cancer-associated pathways, including angiogenesis, epithelial mesenchymal transition, mTOR signaling, myc-targets. The low-risk patients had higher immune cell infiltration levels than high-risk group. We also evaluated the response to chemotherapeutic, targeted therapy and immunotherapy in high- and low-risk patients with LUAD. Furthermore, we validated the expression of the eight gene expression in LUAD tissues and cell lines by qRT-PCR. LYSscore signature provide a new modality for the accurate diagnosis and targeted treatment of LUAD and will help expand researchers’ understanding of new prognostic models.

**Supplementary Information:**

The online version contains supplementary material available at 10.1186/s12935-023-03149-5.

## Introduction

Among all cancers, lung cancer is the leading cause of cancer-related death [1]. TNM staging has long been used to predict the prognosis of lung cancer patients. However, tumor heterogeneity can lead to a difference in survival rates in patients of the same stage [2]. Therefore, the development of a biomarker for lung cancer is urgently needed. Moreover, although significant advances have been made in immunotherapy for advanced lung cancer, clinical biomarkers are still needed to identify which patient populations are likely to benefit from the treatment [3, 4].

Lysosomes are key degradative compartments that maintain protein homeostasis. Over 70 rare genetic disorders are caused by their dysfunction, collectively known as lysosomal storage disorders [5, 6]. Previous studies considered lysosomes as a static organelle specialized in processing and recycling cellular waste, but in recent years several studies have shown that lysosomes can change morphology and function in the cytoplasm and ultimately participate in the development of diseases, including metabolic disorders, neurodegenerative diseases, and cancer [6–8]. Several studies have shown that certain types of cancer, including pancreatic, lung, breast, and prostate cancers, as well as glioblastoma and melanoma, rely on lysosomal–autophagic degradation and recycling to scavenge nutrients [9–11]. For instance, AP1S and HSP70 mediated lysosomal degradation of EGFR inhibits tumor reprogression and increases the sensitivity to chemo- and target sensitivity in lung adenocarcinoma [12, 13]. The loss of MTSS1 in lung adenocarcinoma can promote immune escape by reducing AIP4-mediated PD-L1 monoubiquitination and lysosomal degradation [14]. Moreover, the alteration of lysosomal function is also closely related to the tumor microenvironment and immunotherapy of tumors [15, 16]. However, no synergistic effects of multiple lysosome-related genes on lung cancer have been reported. Therefore, it is important to build lysosome-related signature to evaluate lysosome function in lung adenocarcinoma.

In this work, by using the nonnegative matrix factorization (NMF) algorithm, lysosomes-related molecular subtypes were identified based on the TCGA-LUAD cohort. We compared the differences between two clusters in enriched function and TME. Then we explored the significant prognostic values of differentially expressed genes for LUAD patients. Based on these genes, we constructed an eight-prognostic signature using LASSO Cox regression and multiple Cox regression models from the TCGA. The ability of prediction for survival probability were assessed in different GEO datasets. Finally, a nomogram score system combined with risk score and clinical characteristics were built to quantify survival probability.

## Methods and materials

### Data sources and preprocessing

The bulk RNA-sequencing profiles (FPKM normalized), corresponding clinical information, mutations and copy number alterations of lung adenocarcinoma patients were downloaded from TCGA database (https://portal.gdc.cancer.gov/cart) as a training cohort, which contains 59 normal tissues and 535 LUAD tumorous tissues. External validation datasets were obtained from GEO database (https://www.ncbi.nlm.nih.gov/geo/), including GSE50081 (n = 127), GSE72094 (n = 398) and GSE41271 (n = 184). The immune inhibitor treatment cohort IMvigor210, which investigated atezolizumab in metastatic urothelial carcinoma was downloaded from http://research-pub.gene.com/IMvigor210CoreBiologies through the R package IMvigor210CoreBiologies. Single-cell RNA sequence data from the GSE149655 (n = 2) was employed to reveal cell category and the correlation between single cell and the risk model in LUAD. The lysosomes-related genes were downloaded from MSigDB database (https://www.gsea-msigdb.org), including 1340 genes (Additional file 1: Table S1). To annotate lncRNAs and mRNAs with Perl scripts, we downloaded Genome Reference Consortium Human Build 38 (GRCh38). We excluded patients without complete clinical information and the survival time of 0.

### Screened differentially expressed lysosomes-related genes

First, we screened the differentially expressed genes (DEGs) between tumor and normal samples by “limma” R package according to the filtered criteria (|log2FC|> 1 and FDR < 0.05) in mRNA expression matrix. Then we identified differentially expressed lysosomes-related genes (DELYs) using DEGs list and lysosomes-related genes list through online tool Jvenn (jvenn: an interactive Venn diagram viewer (inra.fr)). By using the “clusterProfiler” and “org.Hs.eg.db” R packages, we investigated the potential biological functions and pathways based on those genes.

### Non-negative matrix factorization clustering analysis for DELYSs

The Non-negative Matrix Factorization (NMF) method based on the standard “brunet” was applied to identify the distinct molecular subtypes of LUAD based on DELYs expression [17]. The R package “NMF” executed this procedure and the samples were iterated thirty times in the TCGA-LUAD cohort. The number of clusters was set as k = 2–10, according to the cophenetic coefficient, contour, and sample size algorithm, and the optimal clustering number was selected as two categories. For evaluating the clinical value of the lysosomes related subtypes with prognosis, Kaplan–Meier survival plot was utilized to compare the OS and PFS of different clusters in TCGA cohort. The relationships of the lysosomes related subtypes with other clinical variables, including immune subtype, survival status, stage and status of lymph node metastasis were visualized by the Sankey diagram drawn using “ggalluvial” R package. The immune subtypes, including C1 (wound healing), C2 (IFN‐γ dominant), C3 (inflammatory), C4 (lymphocyte depleted), C5 (immunologically quiet), and C6 (TGF‐β dominant) (Additional file 1: Table S2) were identified according to Thorsson and colleagues [18].

### The TME evaluation and immune infiltration landscape analysis

“ESTIMATE” algorithm was applied to assess the TME scores, including immune infiltration, stromal score and estimate score of each patient using “limma” and “estimate” R packages. In addition, to explore the immune characteristics of patients with LUAD, we performed single sample gene set enrichment analysis (ssGSEA) based on 23 types of immune cell biomarkers (Additional file 1: Table S3) to quantify the abundance of the immune cell infiltration of each patient. We draw the heatmap to present the differences of immune cell infiltration landscape between clusters and other clinicopathological features such as age, gender, stage, tumor size, status of lymph node and distant metastasis. The “GSVA” and “GSEABase” R packages were utilized to evaluate the potential immune function between two clusters based on the 13 types immune function gene sets (Additional file 1: Table S4). Furthermore, we explored the prognostic values of the immune function for LUAD patients.

### Functional enrichment and TMB assessment

To explore the potential molecular function and pathways, we performed gene set various analysis (GSVA) and GSEA based on defined gene sets, “h.all.v7.4.symbols.gmt”, “c5.go.v7.4.symbols.gmt”, and “c2.cp.kegg.v7.2.symbols.gmt”, which were downloaded from MSigDB database. p < 0.05 was considered to indicate significant differences. The tumor mutation landscape of patients with LUAD was depicted by using “matfool” R package.

### Drug sensitivity prediction

As part of the TCGA cohort, the oncoPredict R package was used to determine the half-maximum inhibitory concentration (IC50) commonly used in chemotherapeutic and targeted drugs for each LUAD patient. There were 198 drugs from Genomics of Drug Sensitivity in Cancer (GDSC; https://www.cancerrxgene.org/) that were compared for sensitivity in the different lysosome clusters. p < 0.05 was set as the threshold for significance.

### Identification of prognostic genes and somatic mutation and copy number alterations analysis in LUAD

To further explore whether these DELYs were associated with LUAD progression, the DEGs were implemented univariate Cox analysis to filter DELYs markedly associated with overall survival (OS) (p < 0.05) using “survminer” package. Thereafter, we depicted the somatic mutation landscape and copy number of the prognostic genes using “matfool” and Perl script. Furthermore, we investigated the proportion of genes alteration types, including mutation, structural variant, CNV alterations. We also investigated the interaction network among these prognostic genes using “igraph” and “psych” package.

### Construction and validation of lysosomes-related signature and nomogram

To better understand the association between lysosomes related clusters and the prognosis of LUAD patients, we constructed a lysosomes-related prognostic model for prognosis prediction. First, as part of the model selection process, we used the least absolute shrinkage and selection operator (LASSO) of Cox regression. Subsequently, the selected genes were performed multivariate Cox regression analysis by “glmnet” and “survival” package. The lysosomes-related risk model was calculated using following formula: LYSscore = Σ (Expi * coefi), where Coefi represented the risk coefficients, and Expi meant expression value of each gene, respectively. According to the median value of LYSscore, patients were classified as high-risk and low-risk group. The time-dependent receiver operating characteristic (ROC) curves and the area under curve (AUC) were utilized to measure the reliability and stability of the risk model by package “survivalROC”. A similar method was used to validate the model's predictive accuracy on the GEO cohorts. At the same time, we described the changes of survival status and number of patients between different risk groups with the increasing risk score. We compared the differences and the percentages of patients between high-risk and low-risk in multiple factors, including age, gender, clinical stage. What’s more, using univariate and multivariate Cox regression analysis, we examined whether the risk score was an independent indicator of prognosis in patients with LUAD. In addition, we drew the Sankey diagram to find the relationships between risk groups and lysosomes-related cluster, immune subtype, and survival status of patients. To improve the accurate predictive power for LUAD patients, we combined clinicopathological features with risk score to construct a nomogram score system. Nomogram was constructed using the "rms" package to predict 1-, 3-, and 5-year survival. The accuracy of the nomogram was validated by calibration, ROC, and decision curves (DCA) using “ggDCA” and “survival” package, respectively. Furthermore, to test the superiority of the risk model, we compared our risk score with other signature, including Wang et al. [19], Jiang et al. [20], Deng et al. [21], Huang et al. [22], and Li et al. [23]. The “survcomp” package was used to assess index of concordance (C-index) and RMS.

### Comprehensive analysis of ICI therapy in different risk group

Tracking the Tumor Immune Dysfunction and Exclusion (TIDE) (http://tide.dfci.harvard.edu/), a comprehensive analysis platform based on the tumor expression matrix was used to find biomarkers to predict the effect of immune checkpoint inhibition therapy. The associations of common immune checkpoint expression between different risk groups were explored. To precise describe the immunotherapeutic response to patients in different risk groups, we downloaded the immunophenoscore (IPS) from The Cancer Immunome Atlas (TCIA) (https://tcia.at/home) to predict responses to immune checkpoint blocked. In order to calculate the IPS, MHC molecules, immunomodulators, effector cells (ECs) and suppressor cells (SCs) were considered. It included four types of scores, ips_ctla4_pos_pd1_pos, ips_ctla4_pos_pd1_neg, ips_ctla4_neg_pd1_pos, and ips_ctla4_neg_pd1_neg, to better predict the efficacy of anti-CTLA-4 and anti-PD-1 antibodies. We also validated the predictive value of risk scores for immunotherapy using the IMvigor210 cohort.

### Identification of risk genes by scRNA-seq analysis

The GSE149655 dataset including two purification LUAD tissues (GSM4506699 and GSM4506701) with log normalized RNA expression matrix were converted scRNA-seq data into Seurat objects using the “seurat” R package. Then we performed quality control the scRNA-seq data to exclude low-quality or biased cells according to the criteria: the threshold at cell counts > 3%, cells with the number of genes mapped > 50%, < 5% mitochondrial genes and > 50% at featured RNAs. After this, 1546 cells were for subsequent analysis. Based on the top 1500 highly variable genes, the principal component analysis (PCA) was used to performed for dimensionality reduction, and the top 15 principal components were selected for cell clustering analysis. After this, T-distributed stochastic neighbor embedding (t-SNE) was employed to visualize cell subpopulations in a two-dimensional space using tSNER package, and “SingleR” package was applied to annotate each subpopulation by corresponding featured genes [24].

### Tissue samples collection, cell culture and real-time PCR

Nine pairs of tumor and adjacent non-tumor tissues were collected from LUAD patients who underwent thoracic surgery in the first affiliated hospital of Xi’an Jiaotong University between September 2022 and October 2022 and stored them in liquid nitrogen. Informed consent was obtained from each patient, and the study was approved by the Ethics Committee of the first affiliated hospital of Xi’an Jiaotong University. Normal pulmonary epithelial cells BEAS-2B and LUAD cell lines (A549 and PC9) were purchased from the American Type Culture Collection (Manassas, USA). BEAS-2B and A549 cell lines were cultured in DMEM medium (Gibco, Rockville, USA), and PC9 cell line was incubated RPMI 1640 medium supplemented with 10% fetal bovine serum (Gibco) and 100 U/mL penicillin under a suitable condition (5% CO_2_, 37 °C).The total RNA was extracted from the tissues and cells using an RNA extraction kit (RNAfast200, fastagen, China) according to the manufacturer’s protocol and performed reverse transcription to cDNA using Prime Script RTase (Takara, China) following the protocol. Based on the manufacturer’s instructions, real-time PCR was used to measure mRNA expression levels using SYBR green (Takara, China). The list of the eight genes’ primers used for real-time PCR was provided in Additional file 1: Table S5.

## Results

### The baseline characteristics of patients with LUAD from TCGA and GEO databases

The TCGA-LUAD cohort (including 490 patients) was considered as training cohort in this study. Furthermore, three independent GEO-LUAD cohorts (including 709 patients) were defined as the testing cohorts. The baseline clinical features of the LUAD patients in training and testing cohorts were provided in Additional file 1: Table S6. Overall, in the training cohort, most patients were over aged 60 years old (67.96%), female (54.29%), diagnosed at early stage I–II (53.27%), stage T1–2 (86.53%), stage N0 (64.69%), and stage M0 (65.71%), while the patients from GEO haven’t sufficient clinical information as training cohort.

### Screening and identification of DELYs

**Fig. 1:**
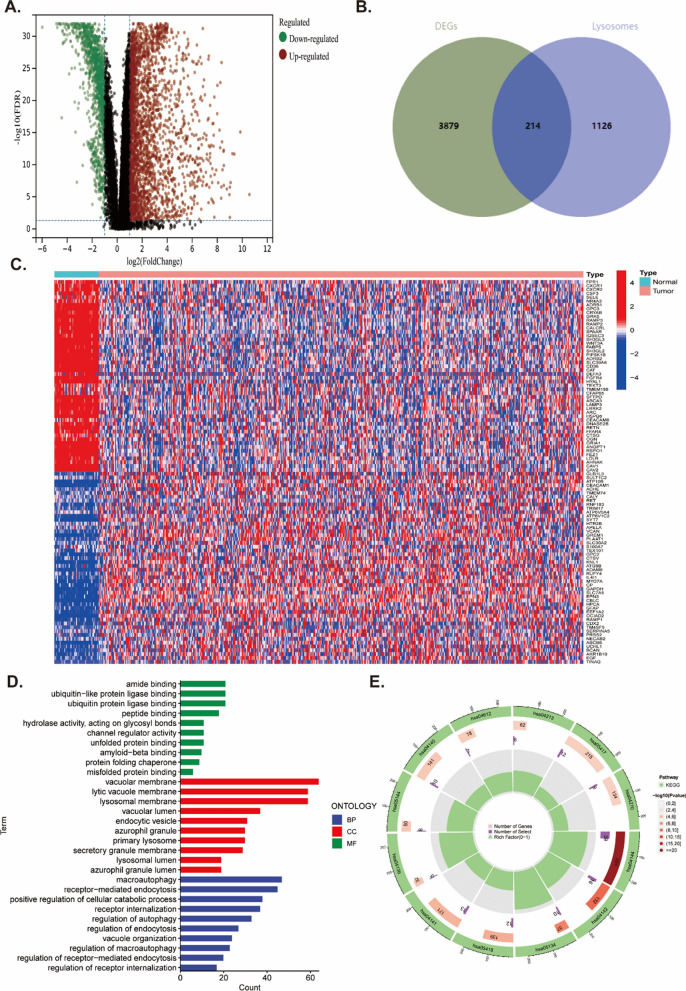
The differentially expressed genes in tumor and normal tissues of patients with LUAD. **A** Volcano plot of differentially expressed genes identified from tumor and normal tissues of LUAD patients. **B** Venn plot of differentially expressed lysosomes-related genes from differentially expressed genes list and lysosomes-related genes. **C** Heatmap of differentially expressed lysosomes-related genes between tumor and normal tissues. **D**, **E** The top significant GO and KEGG terms enriched by the differentially expressed lysosomes-related genes. P-values were adjusted by false discovery rate

A total of 4093 DEGS between tumor and normal tissues were identified through differentially expression analysis, including 2738 highly expressed genes and 1355 low expressed genes in LUAD patients according to the screened criteria: |log FC|> 1 and FDR < 0.05 (Fig. 1A). Then we screened 214 differentially expressed lysosomes-related genes (DELYs) through the DEGs list and lysosomes-related genes list (Fig. 1B). The heatmap showed the expression landscape of DELYs (Fig. 1C). The function enrichment analysis indicated that 214 DEGs were significantly correlated with 1439 GO items and 12 KEGG pathways (Additional file 1: Table S7), and top 30 GO items and top 10 KEGG pathways were presented in Fig. 1D, E. “amide binding”, “vacuolar membrane”, and “macroautophagy” were the most enriched GO keywords. KEGG analysis indicated that “Endocytosis” and “Lysosome” were significant pathways.

### Identification of lysosomes-related subtypes based on DELYs

**Fig. 2:**
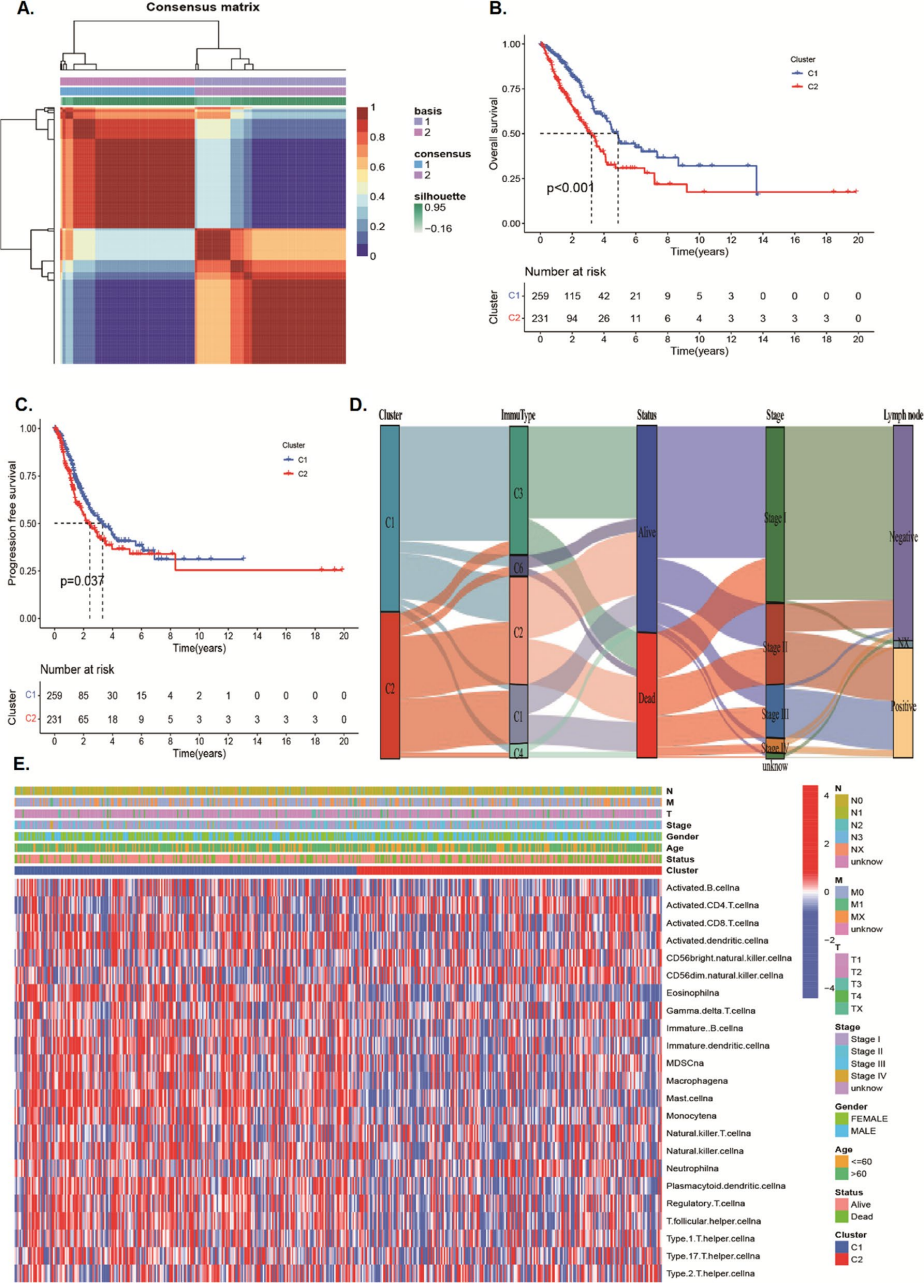
Non-negative matrix factorization clustering analysis for DELYSs. **A** Heatmap of sample cluster when k = 2. **B**, **C** K-M survival analysis of overall survival and progression-free survival for Cluster1 and Cluster2 in TCGA-LUAD dataset, respectively. **D** Sankey plot for patients in different cluster, immune subtype, survival status, clinical stage, and lymph node metastasis status. **E** Heatmap for patients in different cluster with 23 types immune infiltration cells

To further appraise the mechanism of these 214 DELYs in LUAD, we implemented NMF cluster analysis. According to the cophenetic, dispersion, and profile, the K = 2 was considered as the optimal clusters (Fig. 2A, Additional file 2: Figure S1A, B). Patients in cluster2 had poorer overall survival rate and progression-free survival outcomes than patients in cluster1 (Fig. 2B, C). The Sankey plot showed the patients in cluster1 was mainly contributed to immune subtype C3 and had better prognosis (Fig. 2D). To investigate the immune characteristics of patients with LUAD, we drew an immune cell infiltration landscape by comparing the differences of 23 types of immune cell between two cluster and different clinical features. Then we found that cluster1 presented “hot” immune cell infiltration (Fig. 2E). The cluster1 was significantly enriched in activated B cells, activated dendritic cells, eosinophils, immature B cells, immature dendritic cells, MDSC, macrophages, mast cells, monocytes, natural killer cells, plasmacytoid dendritic cells, regulatory T cells, T follicular helper cells, and type 1/17 helper T cells, and cluster2 was significantly enriched in CD56dim natural killer cells (Additional file 2: Figure S2A). The “estimate” algorithm verified cluster1 had higher TME scores, including immune score and stromal score (Additional file 2: Figure S2B). The immune function heatmap suggested that cluster1 had higher levels of Type II IFN response, HLA (human leukocyte antigen), and CCR (chemokine and chemokine receptor) (Additional file 2: Figure S3C), and patients in the high activity of these function had better prognosis than low activity, while patients with high activity of Type I IFN response and para-inflammation had poor survival (Additional file 2: Figure S2D-H).

### Function enrichment analysis and drug sensitivity in subtypes

**Fig. 3:**
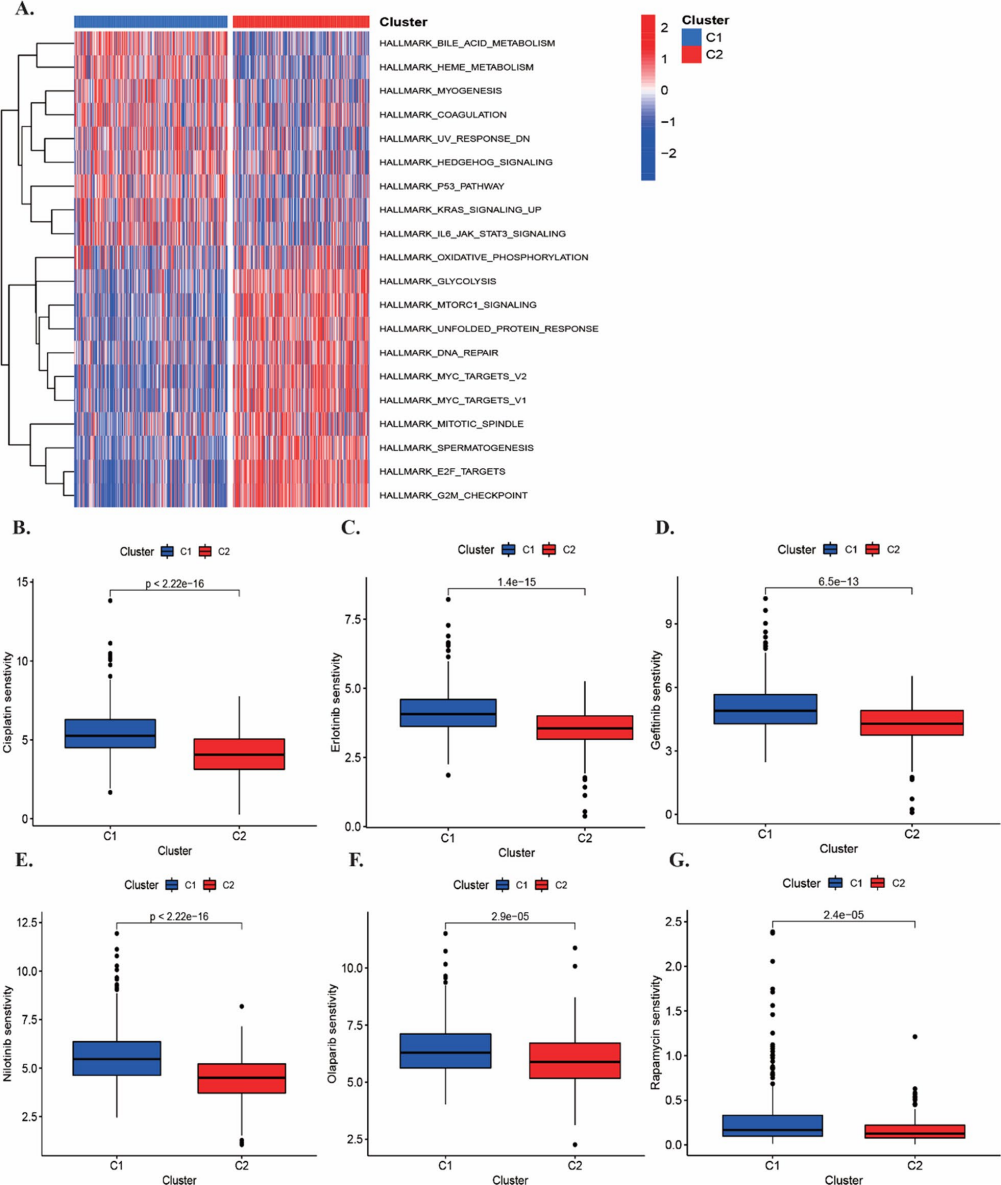
Function enrichment analysis and drug sensitivity in subtypes. **A** The heatmap of GSVA analysis based on Hallmark gene set between cluster1 and cluster2. **B**–**G** The estimation of IC50 indicated the efficiency of chemotherapy and targeted therapy to two subtypes

To elucidate the underlying biological pathways, we performed GSVA analysis of different subtype samples using the defined gene sets and found the correlation with various cancer-related pathways including glycolysis, mTOR targets, DNA repair, myc-targets in cluster2 (Fig. 3A, Additional file 2: Figure S3A, B). The drug sensitivity analysis suggested that cluster2 had lower IC50 in response to chemotherapeutic and targeted drugs including cisplatin, erlotinib, gefitinib and nilotinib (Fig. 3B–G).

### Screened prognostic genes and explicated the landscape of somatic mutation and copy number variation of prognostic genes in LUAD

**Fig. 4:**
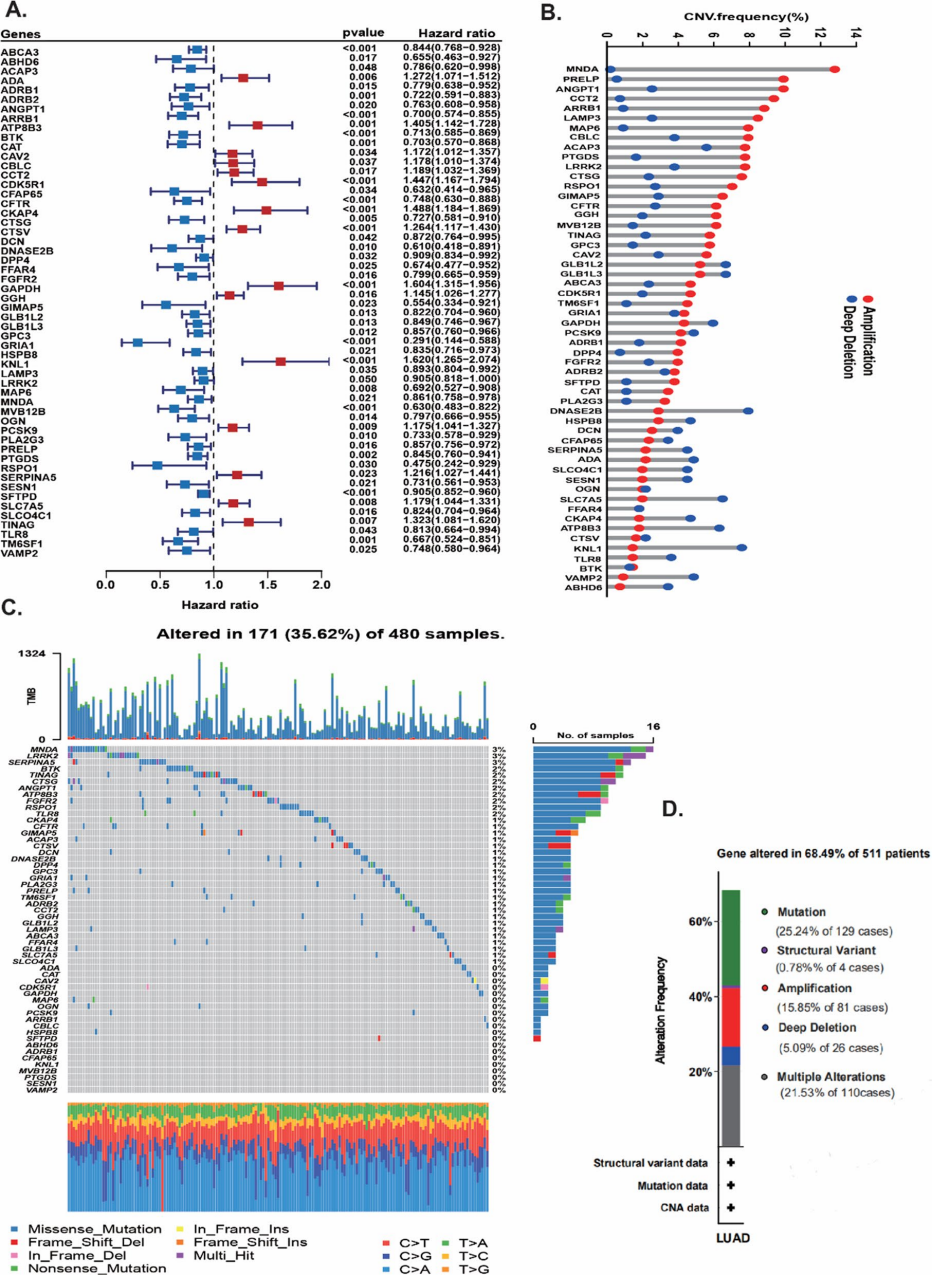
Screened prognostic genes and explicated the landscape of somatic mutation and copy number variation of prognostic genes in LUAD. **A** The forest plot of 54 prognostic DELYs in LUAD patients. **B** The gain or loss status of copy number variation of 54 prognostic genes in LUAD patients. **C** The somatic mutation landscape of 54 prognostic genes in LUAD patients. **D** The proportion of patients with different gene alterations

In this study, we identified 54 prognostic DELYs in LUAD patients (Fig. 4A). Among 54 survival genes, 39 survival genes were protective genes (HR < 1), and 15 genes were related to poor survival (HR > 1). We explored the somatic copy number variation of 54 genes, and we discovered that most of DELYs had CNVs amplification. Of them, MNDA, PRELP, ANGPT1, CCT2, ARRB1, LAMP3, MAP6 showed widespread CNVs amplification, while some of the prognostic genes had CNVs depletion, including DNASE2B and KNL1 (Fig. 4B). We further investigated the mutation landscape of the 54 genes, and of the 480 LUAD patients, genetic mutations of prognostic genes were found in 171 samples (35.62%) of 480 patients (Fig. 4C, D). Among these genes, MDA, LRRK2 and SERPINA5 were the genes which had the highest mutation frequency (3%). We also found that missense mutation was the most common variation type, and C > A and C > T ranked the top single nucleotide variation (SNV) class.

### Construction and validation of lysosomes-related genes risk model in LUAD patients

**Fig. 5:**
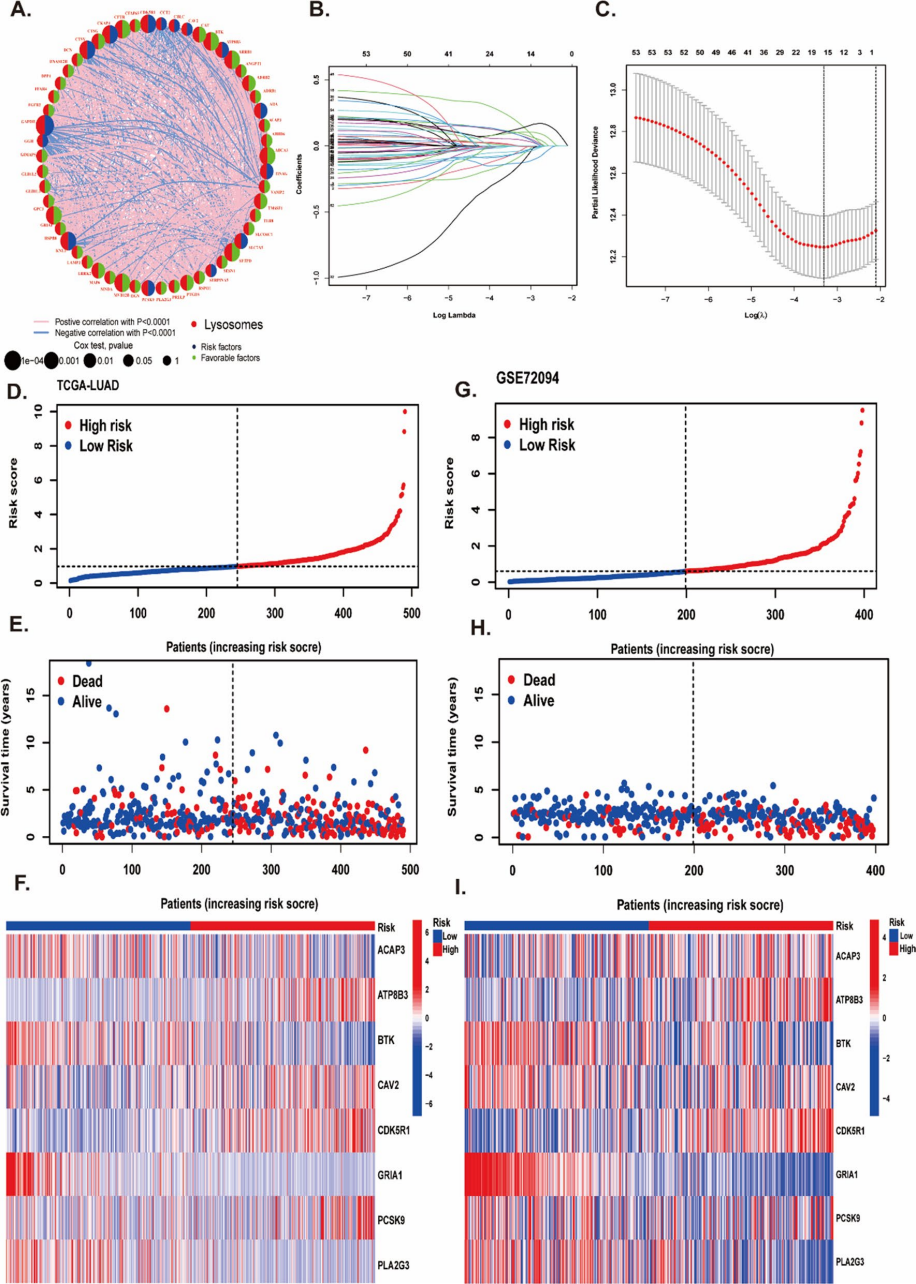
Construction and validation of lysosomes-related genes risk model in LUAD patients. **A** The protein–protein interaction network of 54 prognostic DELYs. **B** LASSO coefficient profiles of the 54 prognostic DELYs. **C** LASSO regression with tenfold cross-validation obtained 17 prognostic genes. **D**–**F** The distribution of risk scores (**D**), survival status (**E**) and genes expression levels of LUAD patients (**F**) in the TCGA cohort. **G**–**I** The distribution of risk scores (**G**), survival status (**H**) and genes expression levels of LUAD patients (**I**) in the GSE72094 cohort

**Fig. 6:**
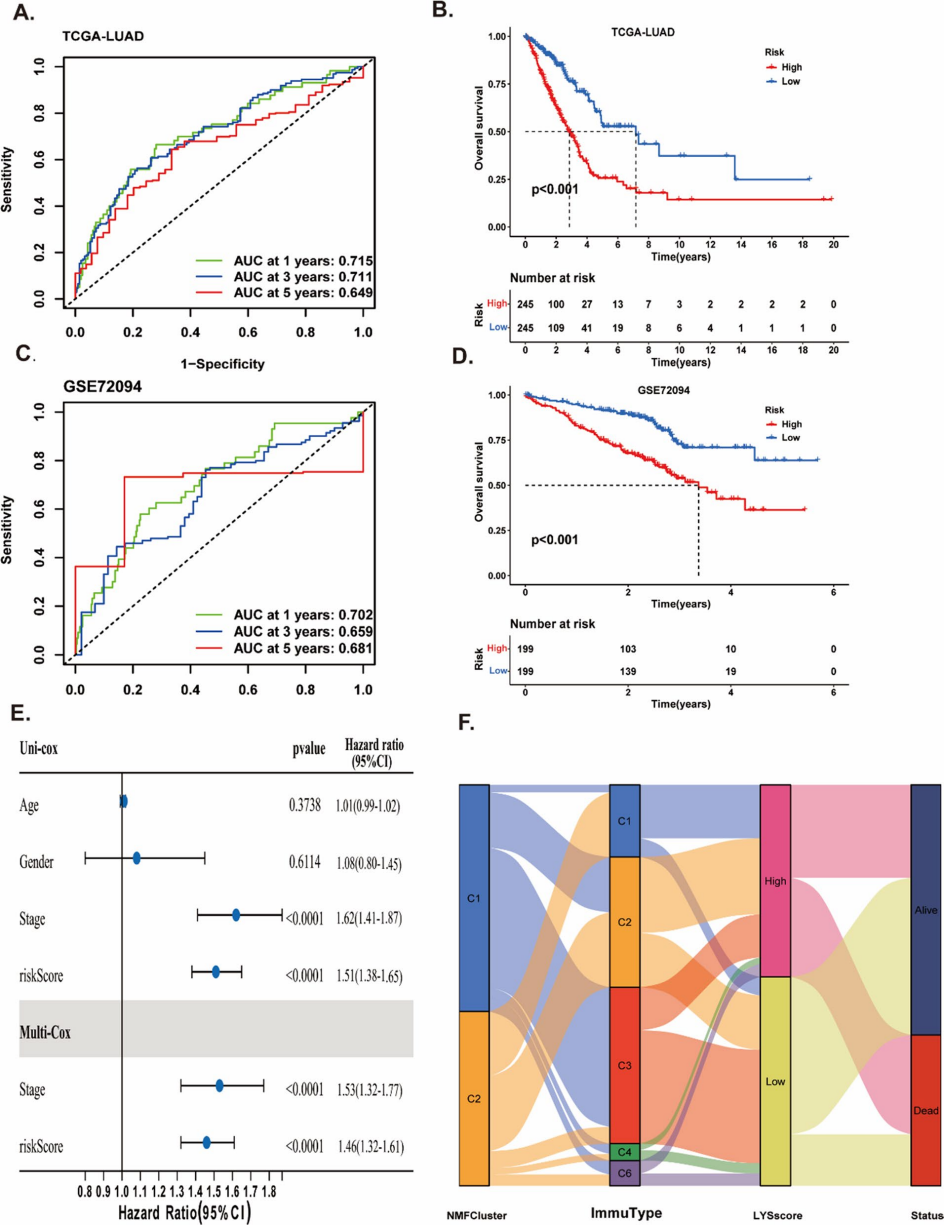
The predictive performance of the prognostic signature in LUAD patients. **A** ROC curve of 1-, 3- and 5-year survival predictions of lysosomes-related signature in the training cohort. **B** Kaplan–Meier survival curves of OS in the training cohort. **C** ROC curve of 1-, 3- and 5-year survival predictions of lysosomes-related signature in the validation cohort. **D** Kaplan–Meier survival curves of OS in the validation cohort. **E** Forest plot of univariate and multivariate Cox regression analyses for the prognosis of LUAD patients in the training cohort. **F** The Sankey plot for two clusters, immune subtypes, two risk groups and different survival status

To find the associations among these genes, we built a protein–protein interaction network. The network showed a strong interaction activity among these molecules at protein level (Fig. 5A). To more directly perceive the prognosis of LUAD patients, we built a predictive prognostic model using LASSO and multivariate Cox regression. After 1000 iterations, we successfully established an eight LYSs signature in TCGA cohort (Fig. 5B, C) (Additional file 1: Table S8). The coefficients of the eight genes (ACAP3, ATP8B3, BTK, CAV2, CDK5R1, GRIA1, PCSK9, and PLA2G3) were presented in Additional file 1: Table S9.The risk score was calculated as following formula: LYSscore = (− 0.3041 * expression of ACAP3) + (0.1975 * expression of ATP8B3) + (− 0.3257 * expression of BTK) + (0.1671 * expression of CAV2) + (0.4774 * expression of CDK5R1) + (− 0.8825 * expression of GRIA1) + (− 0.2217 * expression of PCSK9) + (− 0.1657 * expression of PLA2G3). Based on the median of risk score, the patients were divided into high risk (n = 245) and low-risk group (n = 245), and the clinical characteristics of patients in high or low risk group were presented in Tables S10. What’s more, we investigated the scatters of risk score, survival status, and risk gene expression in both TCGA (Fig. 5D–F) and GSE72094 (Fig. 5G–I). These results indicated that the high-risk group had an increasing number of dead patients compared with the low-risk group in both the TCGA and GEO databases. What’s more, the LYSscore prognostic signature revealed that high sensitivity and specificity for predicting the OS with AUC values of 0.716, 0.711 and 0.649 at 1-year, 3-year, and 5-year, respectively (Fig. 6A). We also compared the AUC value of single gene with signature, and we found the AUC value of the signature better than single gene (Additional file 2: Figure S4). The survival curve showed high-risk group had worse survival rate than low-risk group (Fig. 5B). The stability and reliability of the risk model was validated in GSE72094 (Fig. 6C, D), GSE50081 (Additional file 2: Figure S5A, B) and GSE41271 (Additional file 2: Figure S5C, D) datasets using the same method, and the signature had good predicting performance. Combining with clinical pathological features, we identified the risk score was an independent indicator through univariate and multivariate Cox regression in TCGA-cohort (Fig. 6E). The HR of the risk model was 1.51 (95% CI: 1.38–1.65; p < 0.0001), 1.46 (95% CI: 1.32–1.61; p < 0.0001) in univariate Cox method and multivariate Cox regression, respectively. The Sankey program showed the patients in cluster2 had high risk score (Fig. 6F). In addition, the comparison of risk score in groups with different age, gender and stage subgroups in Additional file 2: Figure S6A–C. Percentage of patients in different risk group were presented in Additional file 2: Figure S6D–F. The male and advanced stage patients had higher risk score, and patients whoever age, gender and stage had poor survival rate. However, there was no significant difference between different age subgroups (Additional file 2: Figure S6G–I). Furthermore, we characterized the genetic variations and expression landscape of eight risk genes based on all the patients from TCGA cohort to understand the CNV status, mutation frequencies and the link to clinical features. The circos plot showed low gain or loss status of CNV variations of eight risk genes (Additional file 2: Figure S7A). Additional file 2: Figure S7B presented that the correlation strength among these genes in LYSscore. The waterfall plot showed few alterations happened in eight genes (Additional file 2: Figure S7C, D). Heatmap showed the relevance between eight risk genes expression and clinical pathologic parameters (Additional file 2: Figure S7E). The detail numbers of patients in different clinical subgroups were displayed in Additional file 2: Figure S8A–D. The performance of the risk model was compared with other signatures, and we found our risk model had better predicting ability and the highest C-index (Additional file 2: Figure S9).

### Clinical value of the prognostic signature

**Fig. 7:**
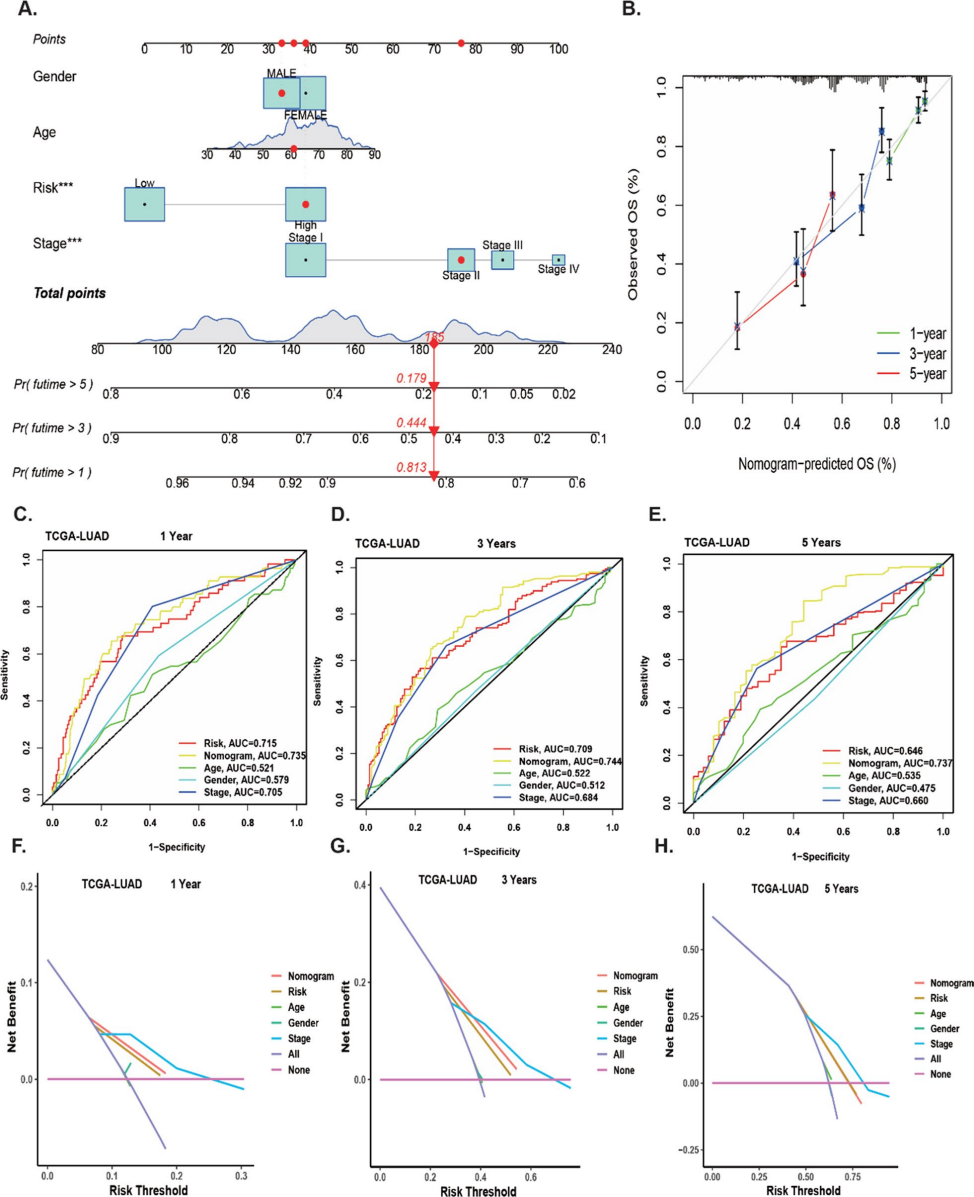
Construction and assessment of the signature-based nomogram. **A** A nomogram constructed based on the TCGA cohort for predicting the 1-, 3-, and 5-year OS of LUAD patients. **B** Calibration curves evaluating the consistency of the actual survival time and nomogram-predicted probability of 1-, 3- and 5-year OS in TCGA. **C**–**E** ROC analyses of the nomogram’s predictive efficacy for 1-, 3- and 5-year OS of patients in TCGA. **F**–**H** Decision curve analysis of the net clinical benefit of the nomogram, the risk score, age, gender and TNM stage for predicting 1-, 3- and 5-year OS of patients in TCGA

To improve the clinical application of prediction model, we constructed a clinically adaptable nomogram score system with the LYSscore and other clinicopathological features to predict the 1-, 3-, and 5-year survival of LUAD patients (Fig. 7A). The nomogram suggested a better accuracy in predicting short survival time. The calibration plot of the nomogram revealed better consistency between the prediction by the nomogram and the actual observation (Fig. 7B). The AUCs of the nomogram at 1-, 3-, and 5-year OS were 0.735, 0.744 and 0.737, respectively, which were better than the risk models and single clinical factors (Fig. 7C–E). Additionally, the DCA curves of the nomogram predicted OS in LUAD patients indicated that this nomogram added more benefit compared with risk model and other clinicopathological characteristics (Fig. 7F–H).

### Characterization of the TME, immunotherapeutic and chemotherapeutic response and in different risk group

**Fig. 8:**
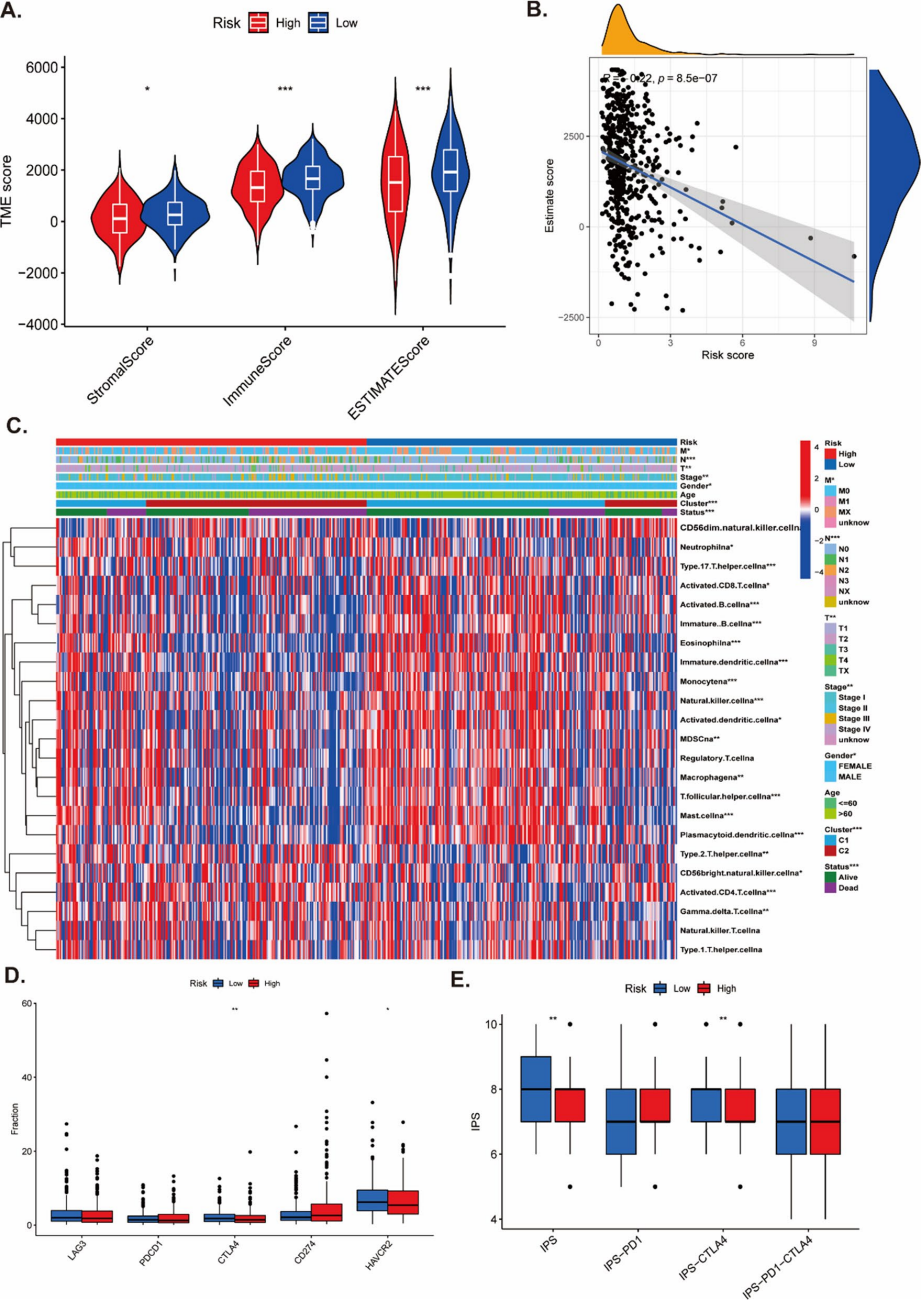
Tumor microenvironment in the high and low risk groups. **A** Comparison of the stromal score, immune score, and ESTIMATE score between high-risk and low-risk groups. **B** The correlation between TME score and risk score. **C** The landscape of immune cell infiltration between high-risk and low-risk groups. **D** The difference of common checkpoint between high and low risk groups. **E** The difference of IPS score, IPS-PD1 score, IPS-CTLA4 score, and IPS-PD1-CTLA4 score between high and low risk groups

**Fig. 9:**
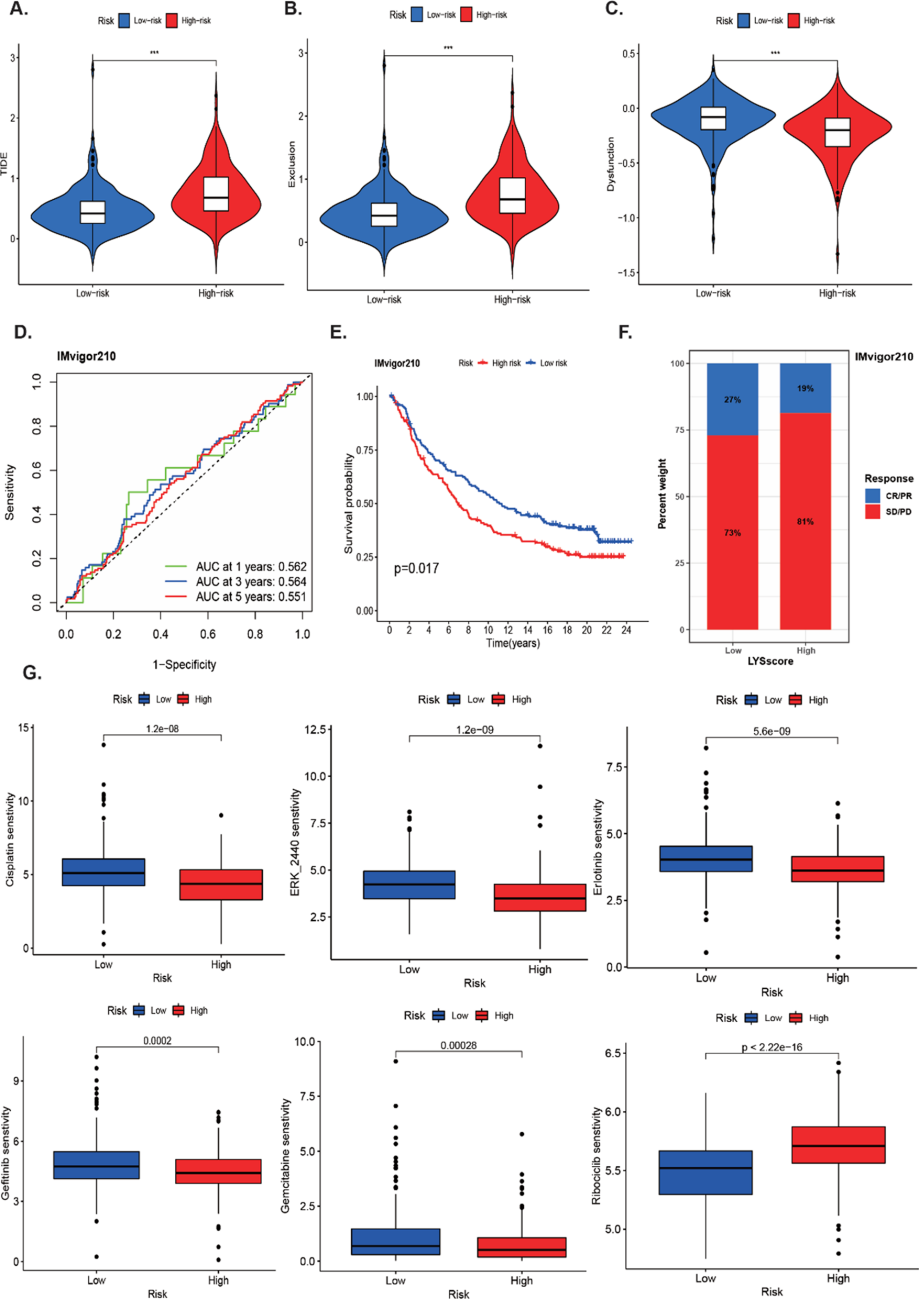
The Characterization of the TME, immunotherapeutic and chemotherapeutic response and in different risk group. **A**–**C** The difference of TIDE score, exclusion score and dysfunction score between different risk group. **D** The ROC curves evaluating the predictive accuracy of the risk score in the IMvigor210 cohort. **E** The high-risk group had poorer prognosis than low risk group in the imvigor210 cohort. **F** The percentage rates of clinical response (complete response [CR]/partial response [PR] and stable disease [SD]/progressive disease [PD]) to anti–PD-L1 immunotherapy in high or low LYSscore groups in the IMvigor210

As lysosomes played an important role in anti-tumor immunity, we explored the association of LYSscore with immune cell infiltration in LUAD patients. By analyzing the TME scores, we found that low-risk patients had higher immune score, stromal score and ESTIMATE score (Fig. 8A). The TME score was negatively correlated with the risk score (Fig. 8B). The immune cells infiltration landscape showed the NK cells, macrophages, MDSC, dendritic cells, monocytes, and eosinophils were markedly enriched in low-risk group (Fig. 8C). The different expression levels of common immune checkpoint (LAG3, PDCD1, CTLA4, CD274 and HAVCR2) between high and low risk group were showed in Fig. 8D, while only CTLA4 and HAVCR2 were significantly marked higher expression levels in low-risk patients compared with the high-risk group. Furthermore, the IPS subtypes (IPS and IPS-CTLA4 score) were higher in low-risk group (Fig. 8E). We also calculated the TIDE, exclusion, and dysfunction score to assess the immune response of LUAD patients. There were significant differences between high- and low-risk groups, and the low-risk patients had lower TIDE and exclusion score, while lower dysfunction score was in high-risk group (Fig. 9A–C). These results indicated that low-risk patients may obtain benefit from immunotherapy. Monoclonal antibodies targeting T-cell suppressor molecules PD-L1 and PD-1 to inhibit immune checkpoints have become an anti-cancer therapy with super survival benefits [24]. Besides, the imvigor210 immune therapy cohort including 298 individuals who accepted anti-PD-L1 treatment was applied to as an external to explore the possibility predictive usefulness of LYSscore. These results revealed that patients with high risk score had a significant poor survival over those with low risk scores (Fig. 9D), while the performance of the cohort was not superior in predicting survival (Fig. 9E). As mentioned in Fig. 9F, 81% patients who had a stable disease/progressive disease (SD/PD) had higher risk score than patients who had complete response/partial response (CR/PR). Finally, in the GDSC database, we looked at the link between chemotherapeutic, targeted therapeutic response and risk scores, and we discovered that patients in the high-risk group were sensitivity to most of drugs, such as cisplatin, ERK240, erlotinib, gefitinib and gemcitabine, while patients with high risk scores were resistant to ribocicib (Fig. 9G).

### Features of the molecular pathways and tumor mutation landscape in distinct risk groups

**Fig. 10:**
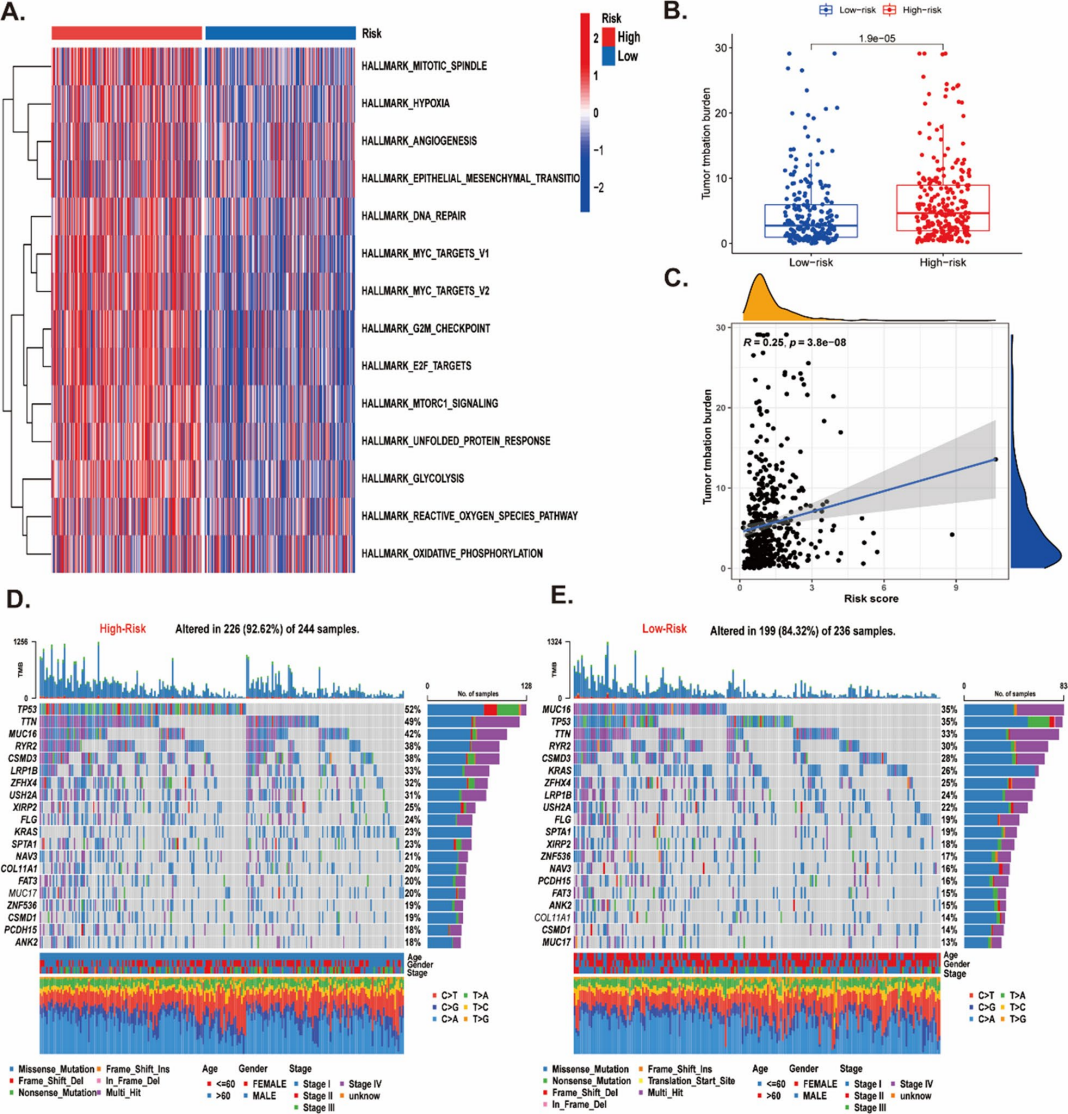
Features of the molecular pathways and tumor mutation landscape in distinct risk groups. **A** The GSVA heatmap showed the differences in pathways in the high and low-risk groups based on the Hallmark gene set. **B** The differences of TMB between high and low-risk groups. **C** The correlation of TMB and risk score. **D**, **E** The tumor mutation landscape showing the mutation status of LYSscore genes was constructed by those with high LYSscore on the left and those with high LYSscore on the right

As presented from the GSVA enrichment analysis, the enrichment of high risk was found in cancer-associated pathways, cell cycle and metabolism, including angiogenesis, epithelial mesenchymal transition, mTOR signaling, myc-targets pathway, G2/M checkpoint, hypoxia, glycolysis, and reactive oxygen species pathway (Fig. 10A, Additional file 2: Figure S10A, B). Subsequently, given the genetic mutations played key role on the tumorigenesis, we used the somatic mutation data to study the situation of mutation between two risk groups. The outcomes displayed that high-risk group had higher TMB than low-risk (Fig. 10B), and the TMB was positive correlation with risk scores (R = 0.25, p < 0.0001) (Fig. 10C). The high-risk group had higher mutation frequency than low-risk group (92.62% (226/244 samples) vs 84.32% (199/236 samples) (Fig. 10D, E). Of these, the missense mutation was the main mutation type, and TP53 was the highest frequency of mutations (52%) in the high-risk group. The primary mutation type in the low-risk group was also missense mutation, while the MUC16 was the highest frequency of mutations (35%). It has been suggested that TMB can be used as a marker to distinguish patients with cancer who might benefit from immunotherapy, and predict the effect of immune checkpoint inhibitors. These results that patients with higher risk scores may were more suitable for immunotherapy.

### The correlation of LYSscores with the single-cell characteristics

**Fig. 11:**
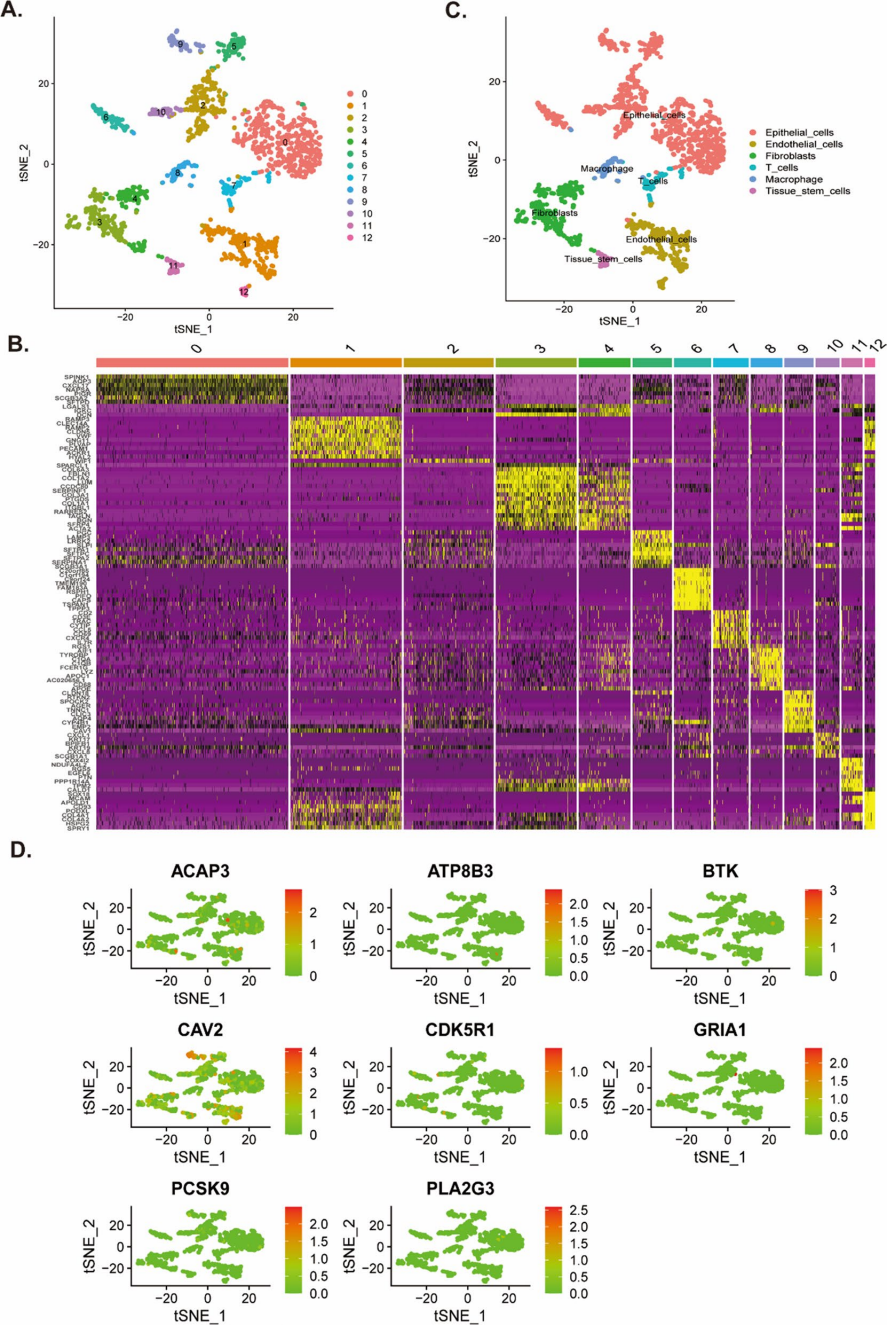
Single-cell RNA-sequencing analysis identified cell types. **A** t-SNE plot of 1546 cells from 2 primary LUAD samples and colored by various cell clusters. **B** The cell types identified by marker genes. **C** Heatmap showing the marker genes in each cell cluster. **D** t-SNE plot of the expression levels of eight prognostic genes

Based on the scRNA-seq data of GSE149655, we obtained gene expression profiles of 1546 cells from two carcinoma samples after initial control (Additional file 2: Figure S11A–S11C). We identified 13 cell clusters by performed PCA using top 1500 variable genes, which reduced the dimensionality (Fig. 11A, Additional file 2: Figure S11D, S11E). The typical genes of each cluster were presented in Fig. 11B. By cross-referencing differentially expressed genes and typical marker genes in each cluster, the annotation of cell identity on each cluster were defined, and cells were mainly annotated six types, including epithelial cells, tissue stem cells, endothelial cells, fibroblasts, macrophages, and T cells (Fig. 11C) (Additional file 1: Tables S11). In order to explore the correlation between risk score and single cells, we depicted the expression density of eight prognostic genes in single cells, and we found just CAV2 presented apparent expression level (Additional file 2: Figure S12A). The expression enrichment of the eight prognostic genes compared to the cell states was shown in Fig. 11D. Furthermore, we analyzed the pseudo-time trajectories of tumor and immune cells in LUAD, and identified three LUAD cell states (Additional file 2: Fig. S12B–S12E).

### Validation of the expression levels of eight lysosomes-related genes in LUAD

**Fig. 12:**
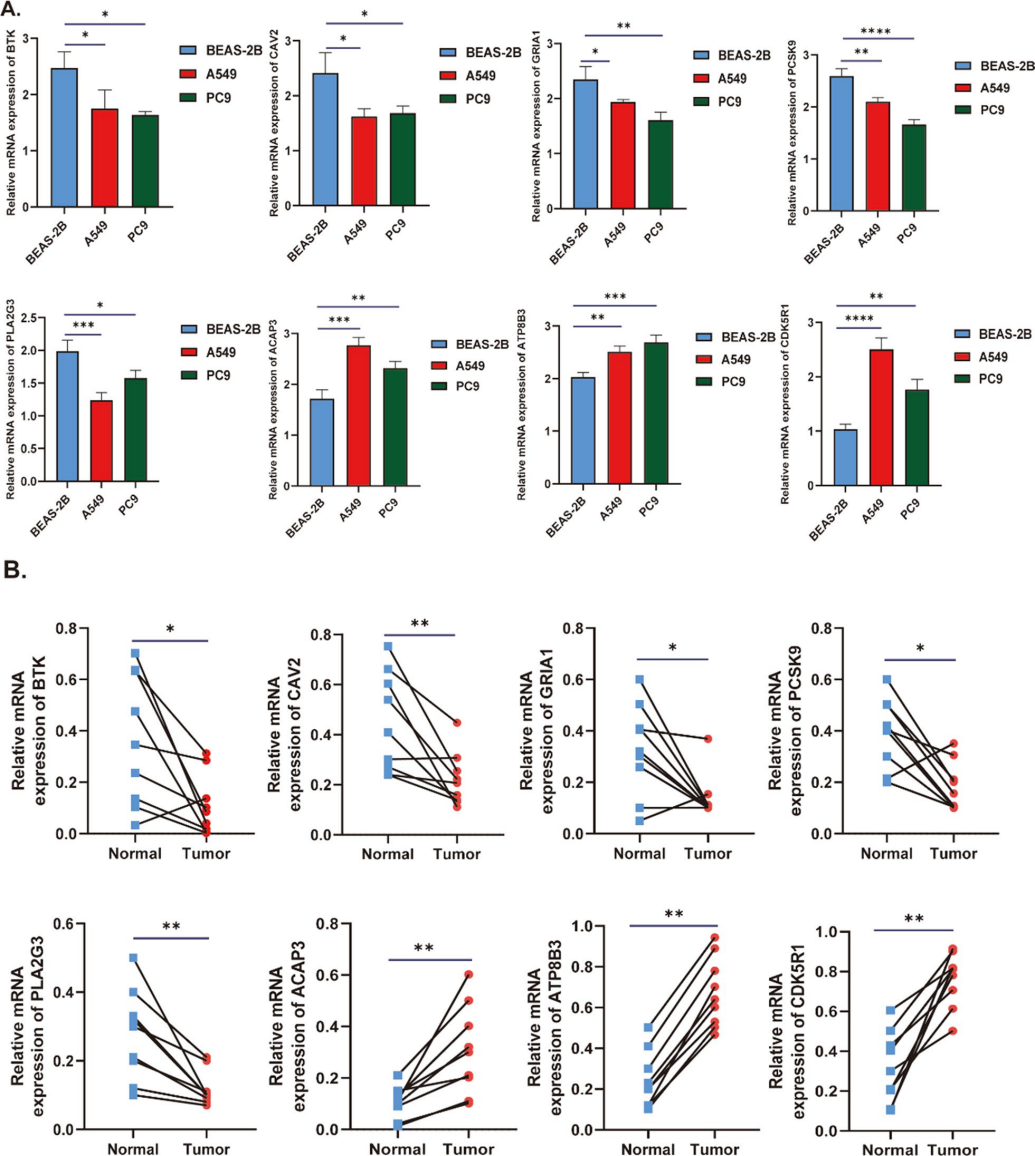
Validation of the expression levels of eight lysosomes-related genes in LUAD. **A** The mRNA expression of eight prognostic genes in BEAS-2B, A549 and PC9 cell lines. **B** The mRNA expression of eight prognostic genes in normal and tumor tissues of LUAD patients

To further verify the expression of these identified prognostic eight genes in LUAD, nine pairs tumor and adjacent nontumor tissues of LUAD patients, and BEAS-2B, A549 and PC9 were used to detect the mRNA expression level of eight genes in this risk score by qRT-PCR. As showed in Fig. 12A, ACAP3, ATP8B3, and CDK5R1 were significantly upregulated in lung adenocarcinoma cells (A549/PC9), while the those of BTK, CAV2, GRIA1, PCSK9 and PLA2G3 were downregulated in A549/PC9 compared to the levels in BEAS-2B. What’s more, we obtained consistent results with previous results our observations in tissues (Fig. 12B).

## Discussion

There has been a long-standing fascination with the lysosome as a potential target for cancer therapy [25, 26]. This was mainly linked to evidence that the lysosome plays a significant role in cell death, as well as its ability to fuel cancer cells' energy needs [6, 27, 28]. Therefore, understanding the role of lysosomes in LUAD will allow for better diagnosis and the development of innovative treatment methods. In our study, based on the expression of 214 DELYs, we divided LUAD into two molecular subtypes, Cluster 1 is more likely to have a favorable outcome. Moreover, cluster1 was significantly enriched in activated B cells, activated dendritic cells, eosinophils, immature B cells, immature dendritic cells, MDSC, macrophages, mast cells, monocytes, and natural killer cells. Previous studies have shown that activated B cells release antibodies and label tumor cells to be recognized and attacked by other cells in the immune system [29], which may suggest a more anti-tumor relationship between lysosomes and the presence of Immune cells. Furthermore, our study found that cluster 1 had higher levels of Type II IFN response, HLA (human leukocyte antigen), and CCR. HLA is a major histocompatibility complex (MHC) product in humans that modulates the immune response to lung cancer by presenting antigens [30, 31]. It has been shown that Type II IFN can directly trigger apoptosis and cell cycle arrest by impairing autophagosome-lysosomal fusion in lung cancer cells [32]. Therefore, this all suggests that DELYs may have an important role in tumor development, which may be related to immune cells and cytokines.

To better understand the mechanisms by which lysosomes affect cancer development, A GSVA analysis was performed using the defined gene sets (KEGG and Hallmark), and glycolysis, mTOR targets, DNA repair, and myc-targets were identified as lysosome-related pathways in cluster 2. Previous studies showed that lysosomal activity may play a role in preserving the quiescence of hematopoietic stem cells by modifying glycolysis-mitochondrial biogenesis [33]. In addition, the mTOR targets, DNA repair, and myc-targets are all involved in the autophagic process and are essential for lysosome formation and transformation [34–36]. This may all suggest that DELYs are closely related to the development of autophagy, which in turn affects tumor development.

Moreover, Mutations in genes are closely related to tumor development, and some mutations have even been used as therapeutic targets [37–39]. However, there is no definitive connection between tumor development and lysosome-related mutations. Our results identified many mutations both in DELYs and related oncogenes, which may be involved in lysosome formation and transformation and could be used as targets for tumor therapy in the future. To better assess the prognosis of lung cancer patients, we also applied lasso regression to analyze lysosome-related genes and found that the model we constructed was superior to previous studies, and superior to tumor markers, such as CEA, CA199, and CA125. To enhance the reliability of this model, we selected GEO dataset as external validation cohort. When selecting datasets for validation, several key factors should be considered: data quality, sample size, diversity, data balancing, availability.

In this study, eight lysosome-related genes (ACAP3, ATP8B3, BTK, CAV2, CDK5R1, GRIA1, PCSK9, PLA2G3) were identified. Some previous studies had illustrated the molecular functions and cellular processes of them. For instance, ACAP3, one of members of the ACAP family of GTPase-activating proteins for the small GTPase ADP-ribosylation factor [40], which are expressed in brain and associated with the endolysosomal pathway [41]. ATP8B3 (belongs to P4-ATPases) is a subfamily member of P-type ATPases that flip phospholipids across membranes to generate lipid asymmetry, which participate in cell component composition [42]. BTK encodes Bruton’s Tyrosine Kinase and plays an oncogenic role on head and neck squamous cell carcinoma, promoting epithelial–mesenchymal transition processes and cancer stem cell enrichment [43]. Research have indicated that Inhibitors targeted to BTK have been developed for hematological tumors [44]. CAV2 is a member of caveolin protein family, which plays a vital role in intracellular cell transport and signal transduction [45]. CAV2 overexpression involves in promoting tumor growth, metastasis and angiogenesis in lung cancer and pancreatic cancer [45, 46]. CDK5R1 is one of the activators of CDK5, which binds and activates CDK5 to drive G1-S transition and RB phosphorylation in medullary thyroid carcinoma models [47]. At present, researchers found that GRIA1 encodes AMPA receptors mediated a fast component of the synaptic current. The variants of GRIA1will contribute to neurological conditions disorder [48]. PCSK9 is a member of the pro-protein convertase family, and plays important roles in proteolytic activation, modification, and degradation of secreted proteins [49]. PCSK9 could clear LDL-cholesterol from the circulation by inducing lysosomal degradation of the low-density lipoprotein receptor in the liver [50]. PLA2G3 is a group of enzymes that hydrolyze phospholipids to release fatty acids (FA) and lysophospholipids regulating lipid metabolism of transformed cells, and the downregulation of PLA2G3 inhibits the tumor growth and promotes chemo-sensitization in ovarian cancer [51]. Furthermore, mast cells could self-secret PLA2G3 to facilitate mast-cell maturation [52]. These eight genes play various function in biological process and cellular functions.

LUAD treatment is being revolutionized by immunotherapy, especially ICI [53, 54]. Nevertheless, due to the heterogeneity of the inter-and intra-tumor tumors, identifying a subpopulation that will benefit from immunotherapy remains challenging [55, 56]. As a result, predictive biomarkers on immunotherapy response and prognosis are crucial to determining LUAD subtypes and improving personalized immunotherapy. Additionally, we have also previously identified a definitive role for lysosomes with immune infiltration. Therefore, we further explored the relationship between LYS score and immune infiltration. Our results indicated that compared to high-risk patients, low-risk patients had higher immune scores, stromal scores, and ESTIMATE scores. Additionally, low-risk patients had enriched NK cells, macrophages, MDSCs, dendritic cells, monocytes, and eosinophils. Our single-cell sequencing validation results were similar and most of these cells are associated with immunotherapy-related processes such as tumor killing and antigen presentation [57–59]. Therefore, this further suggests that lysosomes are closely related to immune infiltration and may serve as an important indicator for assessing the prognosis of immunotherapy. Moreover, our study found that the low-risk group was sensitive to immunotherapy, while the high-risk group was sensitive to drugs like erlotinib and gefitinib, which is consistent with the current findings that people sensitive to EGFR-TKI may not be sensitive to immunotherapy, which suggests that our LYSscore may also be able to further assess patient medication regimens, with important implications for clinical treatments.

Currently, there is no definitive conclusion about how lysosomes modulate immune infiltration and immunomodulators and affect ICI therapeutic response. Our study found that some important pathways that affect tumor development, such as mTOR, angiogenesis, and epithelial-mesenchymal transition, predicted poor tumor immunotherapy outcomes [60–62]. This may suggest a new therapeutic modality that could link mTOR inhibitors, angiogenesis inhibitors, and other related drugs with ICI to produce better therapeutic outcomes in the immunotherapy of LUAD [63].

As compared with existing studies of prognostic signatures for LUAD, this study has several notable advantages as well as limitations. Firstly, our study identified the relationship between lysosome-related genes and lung cancer prognosis and compared it with traditional indicators, establishing a new indicator for lung cancer prognosis assessment that has not been done before and giving some insight into future studies of lysosomes and lung cancer treatment. Secondly, as the first study to analyze the combination of lysosomes and immunotherapy in lung cancer, we developed the LYS score to assess immunotherapy prognosis and proposed new treatment options. At last, we combined single cell sequencing dataset to accurately analyze lysosome-related genes expression at the single-cell level, and we evaluated the performance of this model in predicting immunotherapy response to improve its clinical utility based on the immunotherapy cohort. However, our study still has some shortcomings. Firstly, our studies were mainly analyzed by databases, and although we applied PCR to validate clinical specimens and cell lines, we cannot conduct a more in-depth study due to financial reasons. In addition, the results might be affected by a lack of complete information on surgery and treatment in the database. LYSscore, as a new type of biomarker, faces some challenges in clinical applications. Firstly, the gene expression profile analysis involved in the model may require high experimental conditions and equipment, which may require certain technology and resource investment. Secondly, although LYSscore shows good performance in predicting the response of tumor cells to immunotherapy, whether it is suitable for all types of cancers and patients still needs further research and validation.

In conclusion, we developed a LYS model based on a lysosome gene-guided strategy for predicting LUAD prognosis and immunotherapy efficacy, which has been validated by external transcriptome data and single-cell sequencing data. In addition, by identifying the complex relationship between LYS and oncogenic pathways, such as mTOR, we provided insight into LYS's role in tumorigenesis and TME reshaping. In combination with immune infiltration, immune checkpoint factors, and other biomarkers, we demonstrated that LYS effectively distinguishes responders and non-responders, enabling ICI therapy to be more precisely stratified by benefit. Therefore, this work might facilitate the identification of prognostic biomarkers and provide guidance for developing personalized immunotherapy.

### Supplementary Information

**Additional file 1:** Supplemental tables.

**Additional file 2:** Supplemental figures.

## Data Availability

The datasets generated and analyzed during the current study are available in the TCGA (http://cancergenome.nih.gov/abouttcga) and GEO (https://www.ncbi.nlm.nih.gov/geo/) databases.
