# Supplementary material for: Identification and validation of eight lysosomes-related genes signatures and correlation with immune cell infiltration in lung adenocarcinoma
Source: Cancer Cell Int. 2023 Dec 13;23:322. doi: 10.1186/s12935-023-03149-5 (PMC10720244; doi:10.1186/s12935-023-03149-5)
Supplement: Supplementary file 2 — Additional file 2: Supplemental figures. [file 12935_2023_3149_MOESM2_ESM.docx]

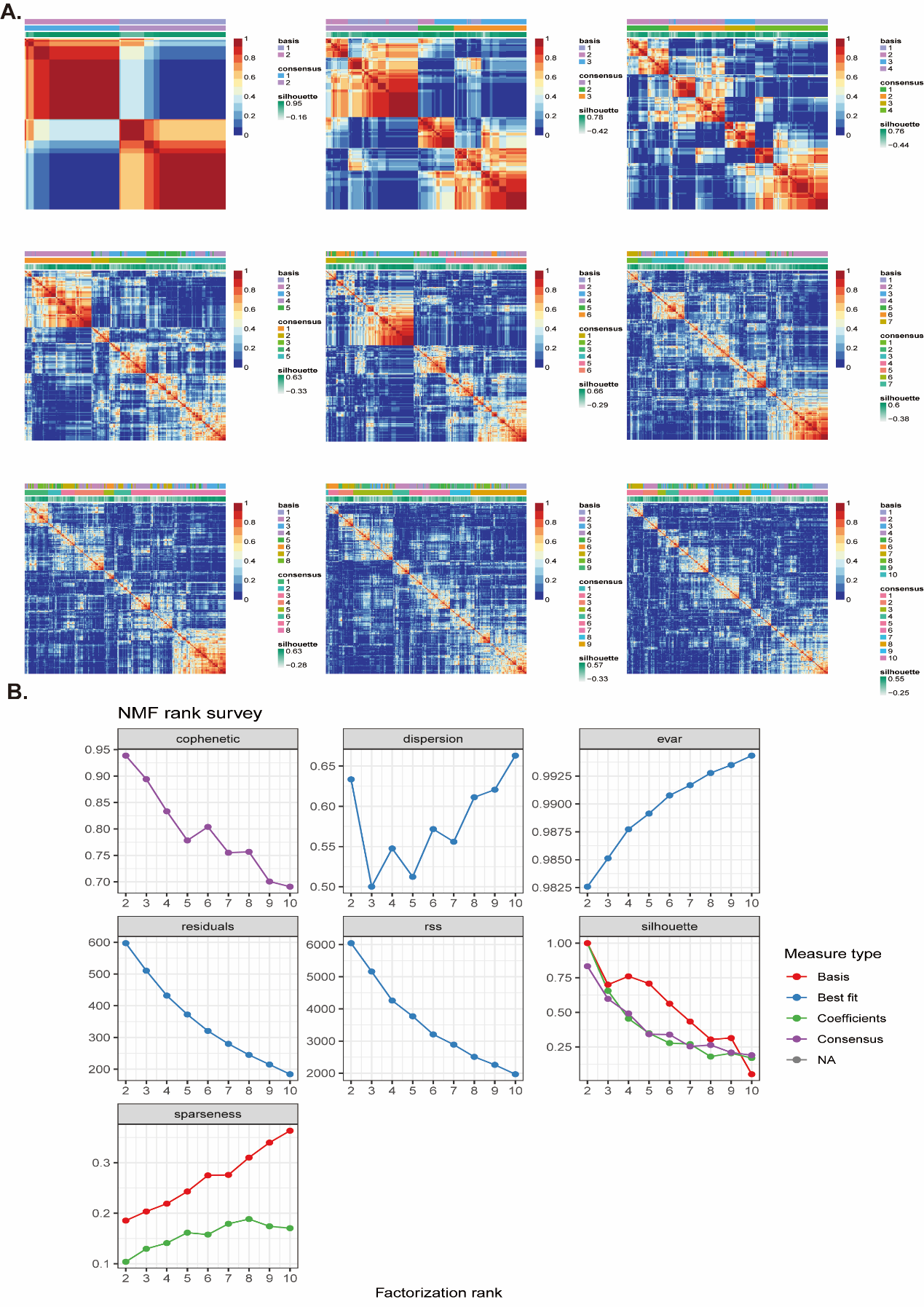


**Figure S1. Non-negative Matrix Factorization of LUAD molecular subgroups based on DELYs.** (A). All the heatmap corresponding to the consensus matrix for k = 2-10 obtained by applying NMF. (B). The curves of cophenetic correlation coefficient, RSS, and dispersion et al. were used to reflect the stability of the cluster obtained from NMF.


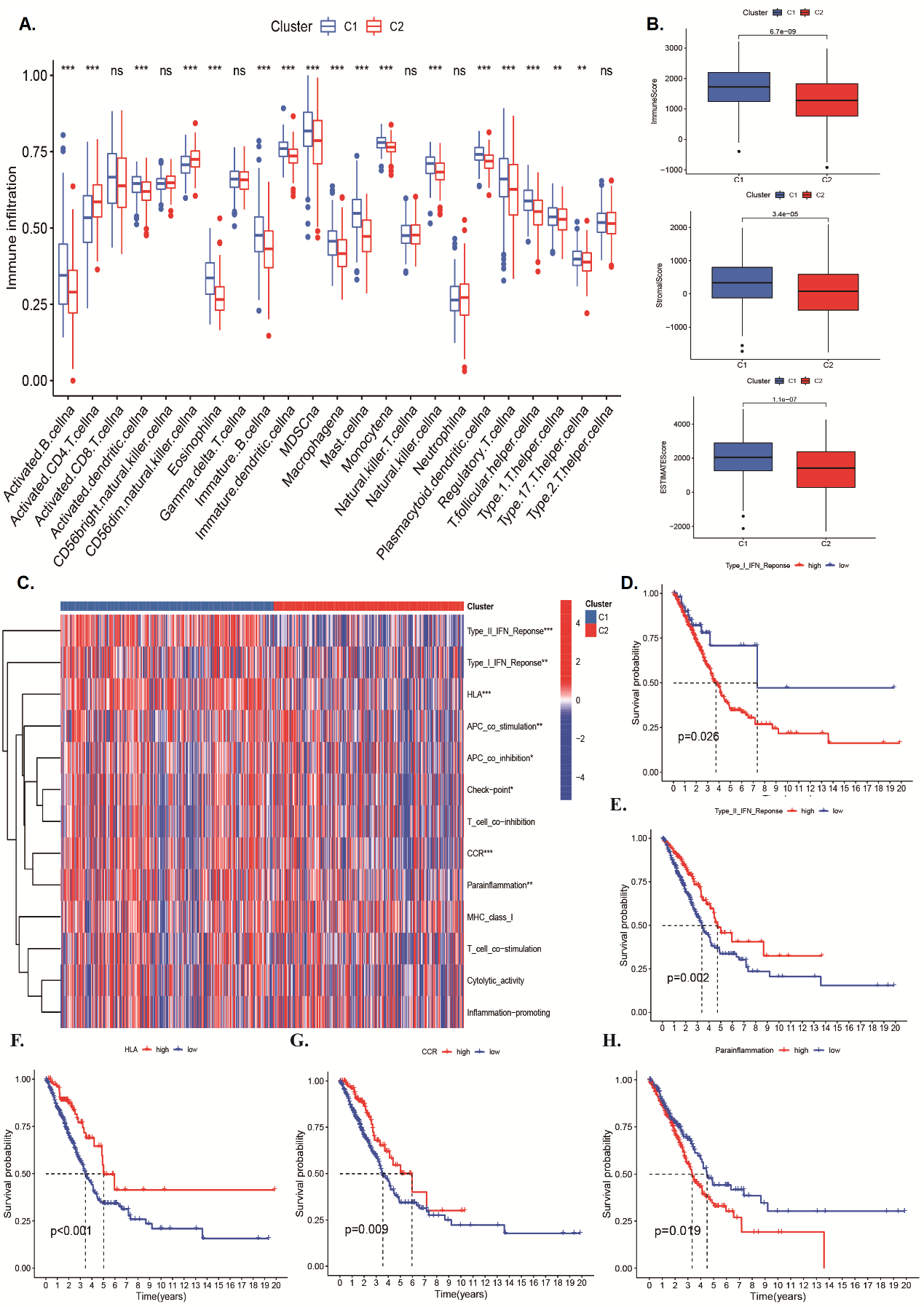


**Figure S2. The TME characteristics of different clusters and prognosis analysis.**

(A). Differences of 23 types immune cell between two clusters. (B). The TME score in different cluster. (C). Heatmap of immune function in two clusters. (D-H). Survival curves of patients with different immune function.


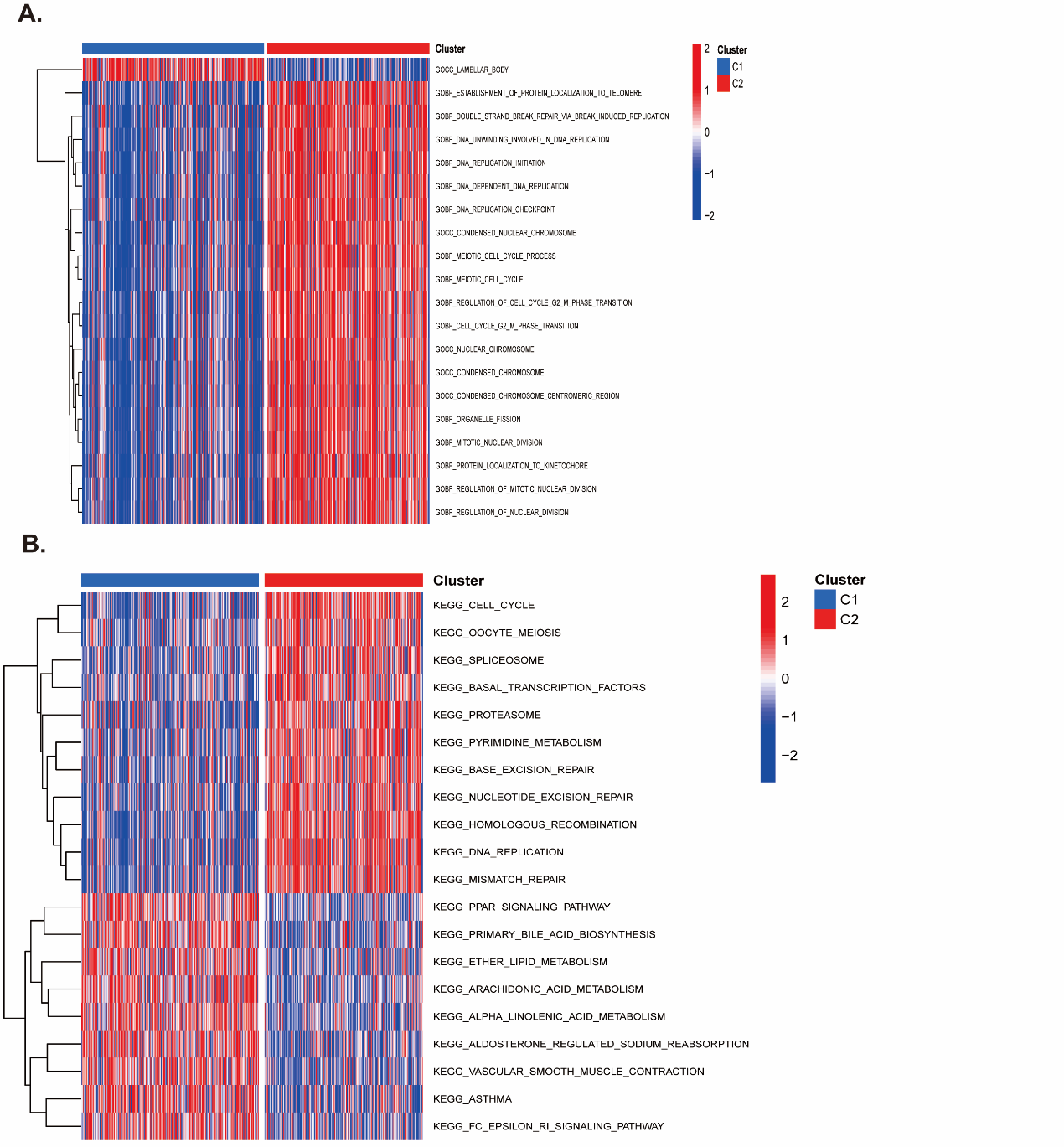


**Figure S3. Function enrichment analysis in subtypes.** (A-B). The heatmap of GSVA analysis based on GO and KEGG gene set between cluster1 and cluster2.


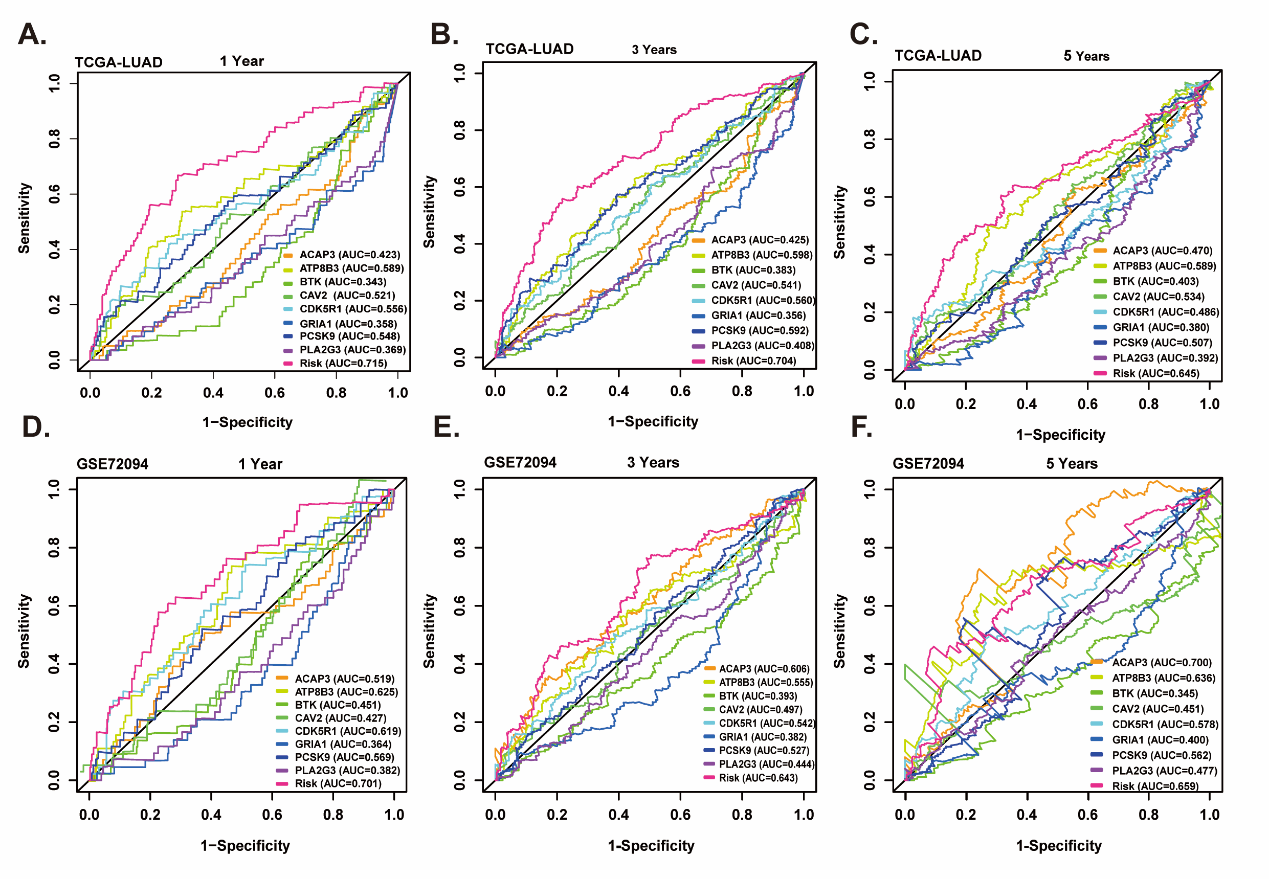


**Figure S4. The ROC curves with respect to eight key prognostic genes in the TCGA cohort and GEO cohort.** (A-C). ROC curve of 1-, 3- and 5-year survival predictions of single gene in the training cohort. (D-F). ROC curve of 1-, 3- and 5-year survival predictions of single gene in the validation cohort.


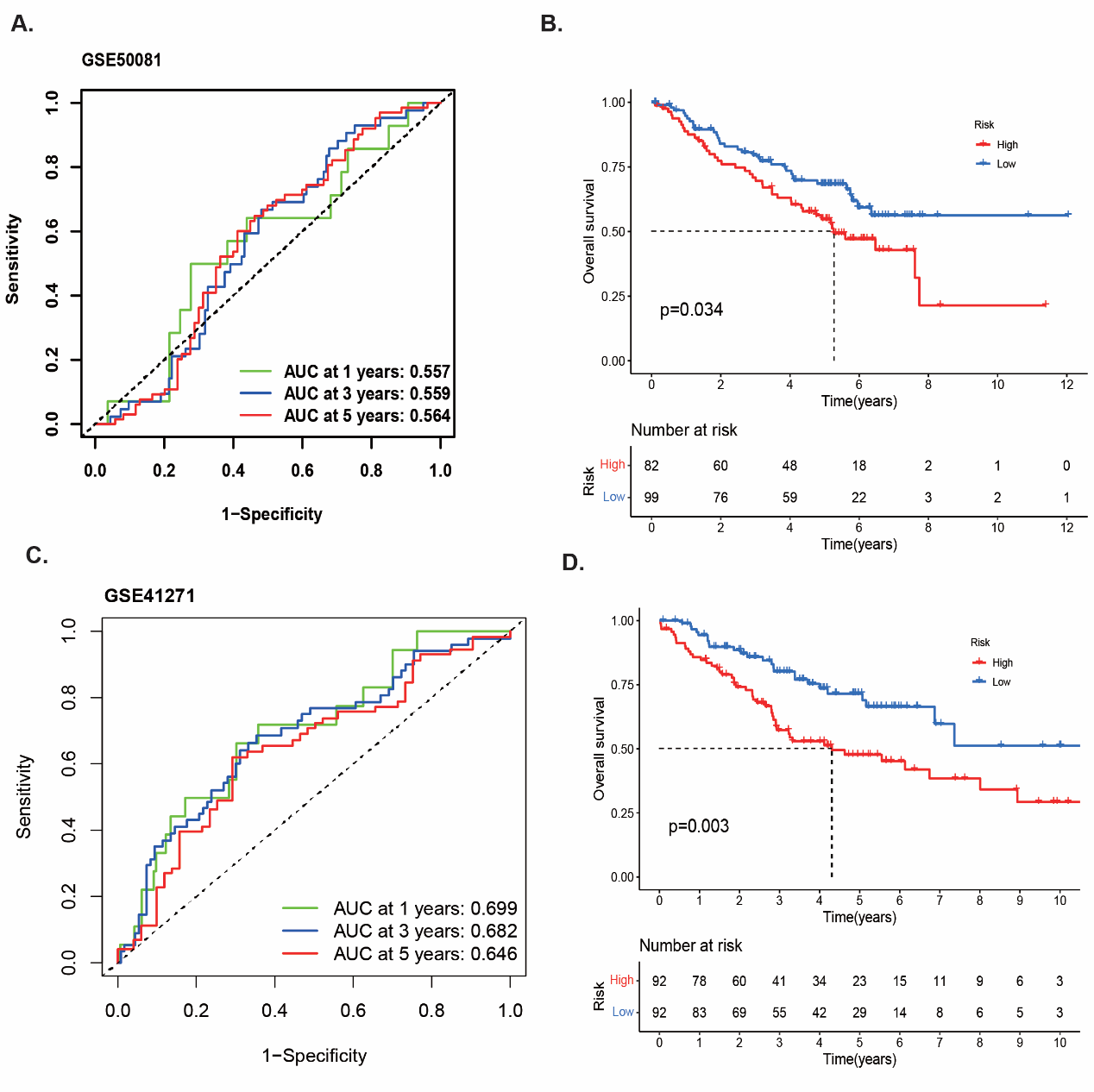


**Figure S5.** **The ROC curves and Kaplan-Meier of the prognostic signature in validation cohort.** (A-B). The ROC curves and Kaplan-Meier of the prognostic signature in GSE50081 cohort. (C-D). The ROC curves and Kaplan-Meier of the prognostic signature in GSE41271 cohort.


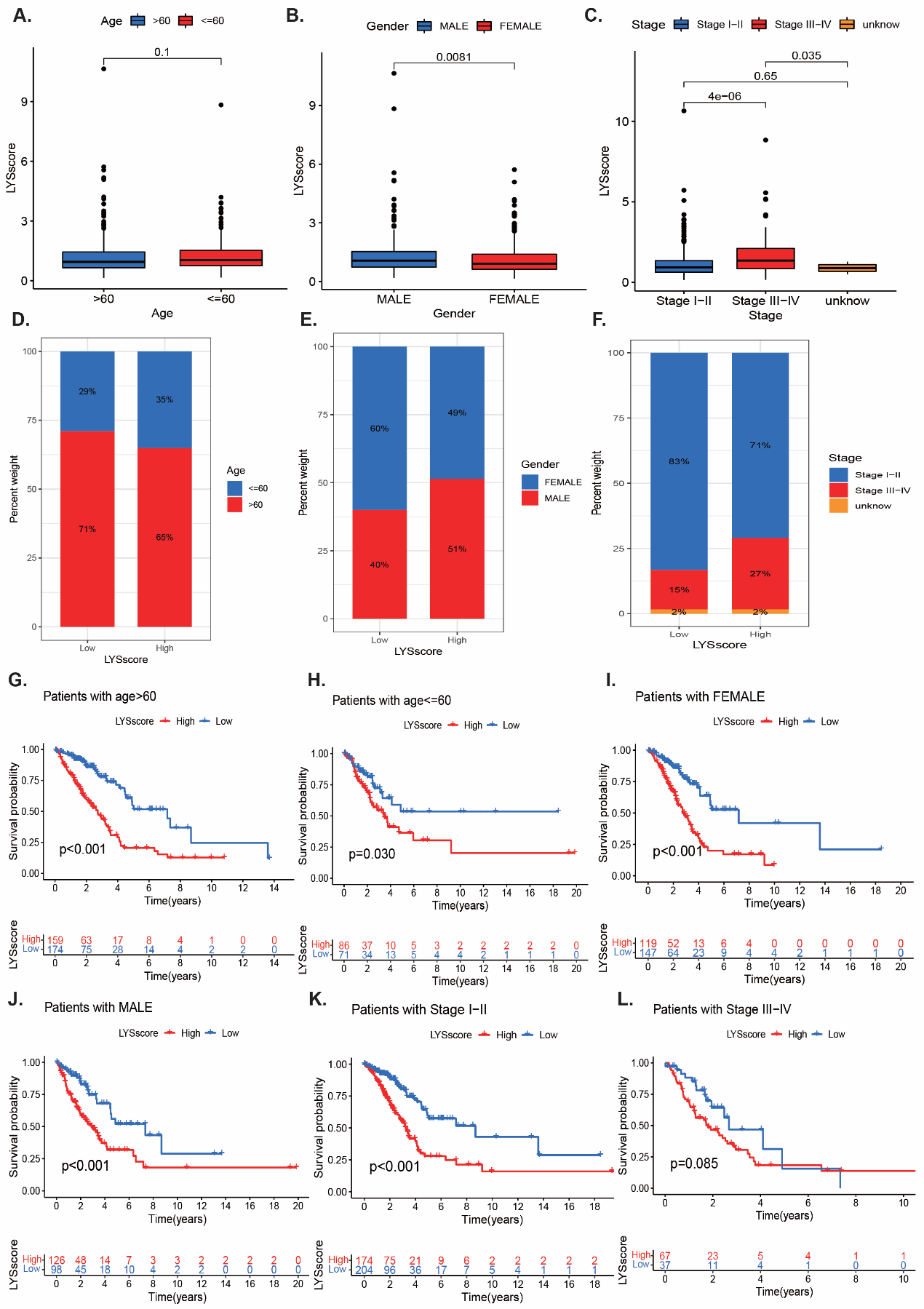


**Figure S6. The clinical characteristics of prognostic signature in TCGA.** (A-C). The differences of risk score in different age, gender, and stage, respectively. (D-F). The percentage of patients with different age, gender, and stage in high and low risk group.

(G-L). Kaplan-Meier survival curves of OS in the different clinical subgroups.


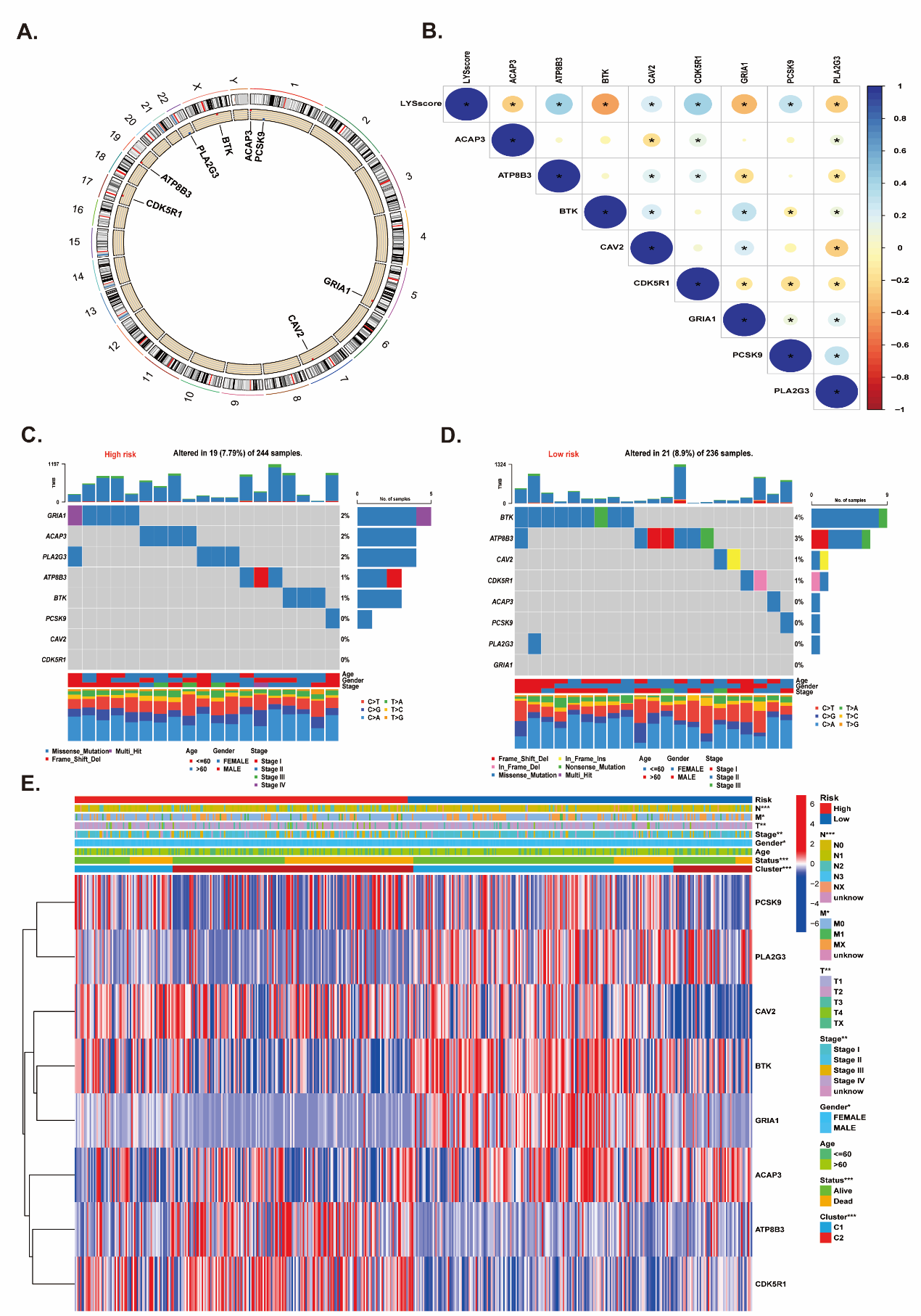


**Figure S7. The characteristics of eight prognostic genes in risk model.** (A). The locations of CNV variations in eight prognostic genes on 23 chromosomes. (B). The correlation between eight prognostic genes and LYSscore. (C). The genetic variations frequencies of eight prognostic genes in high and low-risk group. (D). The expression differences of eight prognostic genes between two risk groups and clinical features.


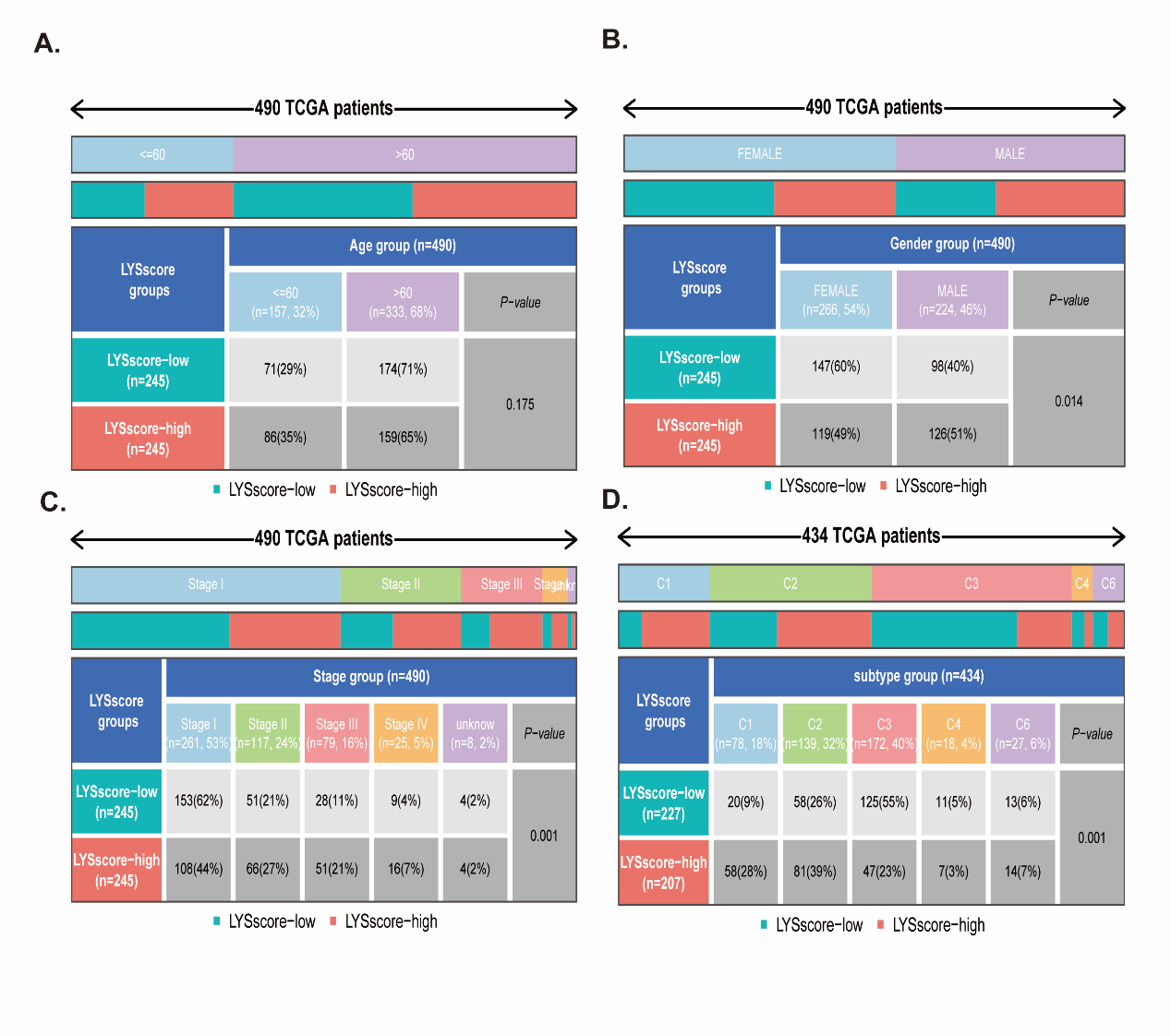
 **Figure S8. Distribution of age, gender, stage and immune subtypes in the different LYSscores groups.** (A). Heatmap and table showing the distribution of different age LUAD patients between the LYSscores groups. (B). Heatmap and table showing the distribution of different gender LUAD patients between the LYSscores groups. (C). Heatmap and table showing the distribution of different stage LUAD patients between the LYSscores groups. (D). Heatmap and table showing the distribution of different LUAD immune subtypes between the LYSscores groups. The distribution of the clinical characteristics and immune subtypes in the LYSscore subtypes were compared through the χ^2^ test.


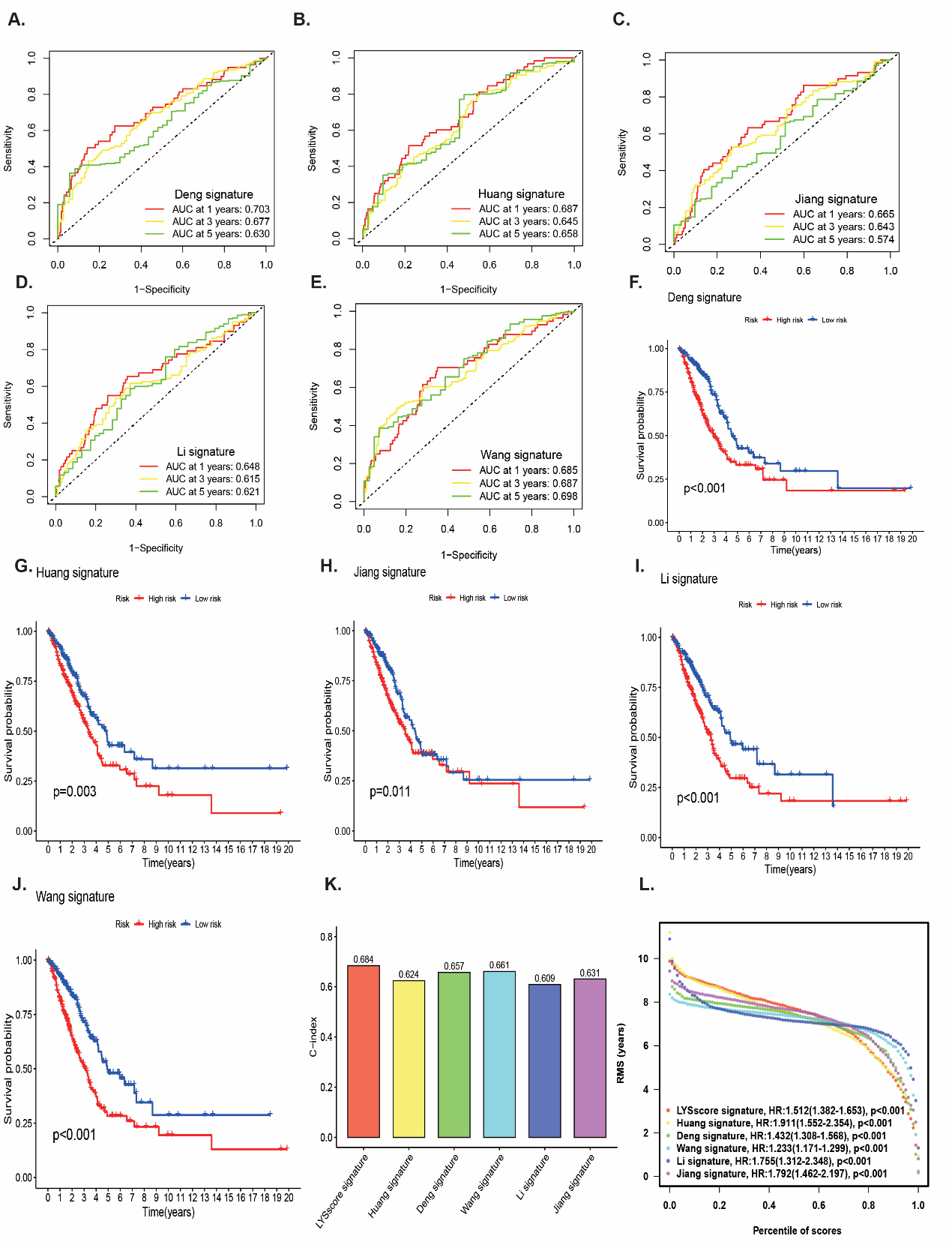
 **Figure S9. Comparisons of ROC curves of the LYSscore signature and other signatures for 1-, 3- and 5-year OS in the TCGA cohort.** (A-E). The ROC curves of Deng, Huang, Jiang, Li and Wang signature for 1-, 3- and 5-year OS, respectively. (F-J). Kaplan-Meier of Deng, Huang, Jiang, Li and Wang signature for 1-, 3- and 5-year OS, respectively. (K). The C-index of LYSscore signature and other signatures. (L). The RMS curves of LYSscore signature and other signatures.


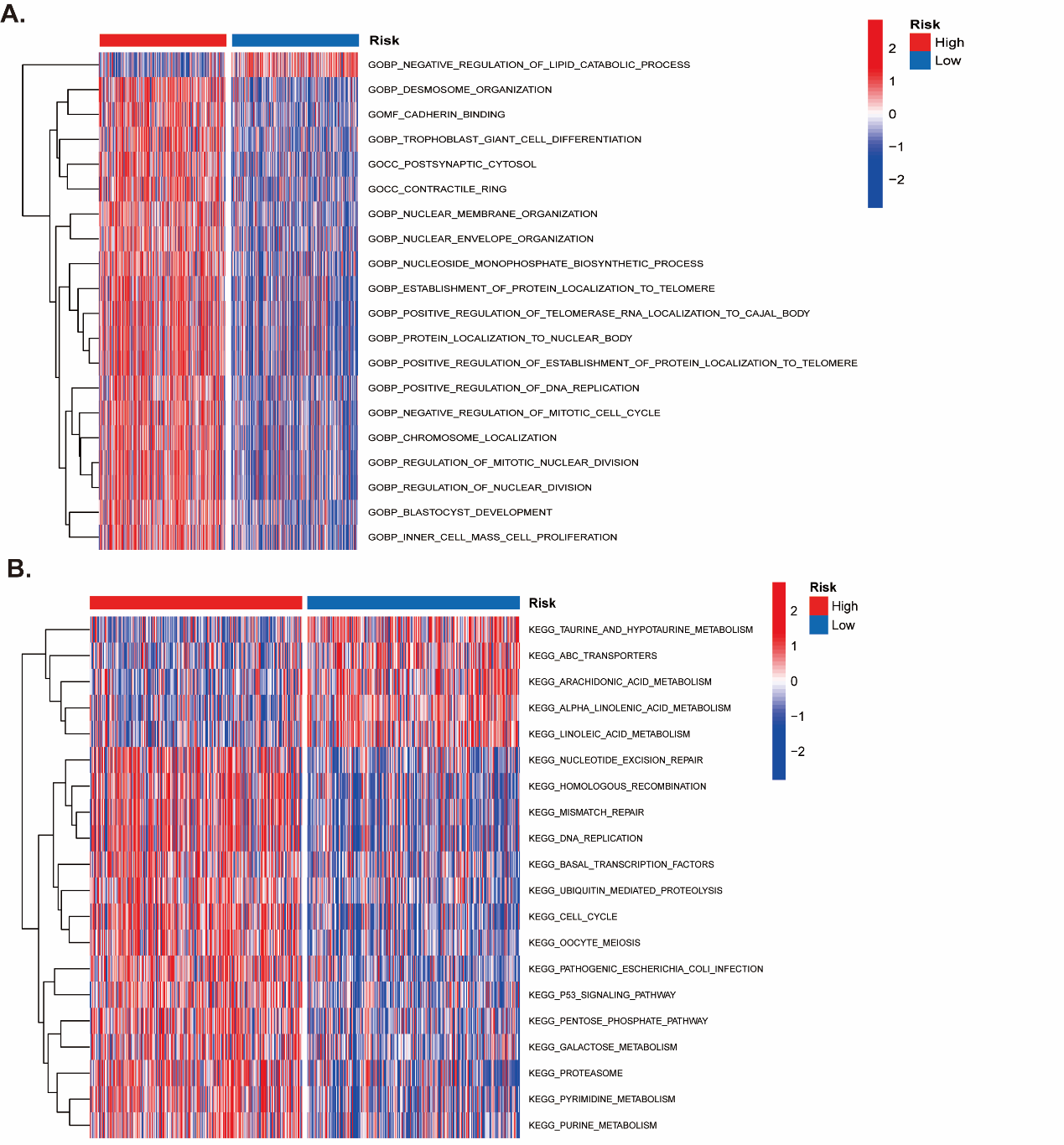
 **Figure S10. The GSVA heatmap showed the differences in pathways in the high and low-risk groups.** (A). GSVA analysis based on the GO gene set and KEGG gene set (B).


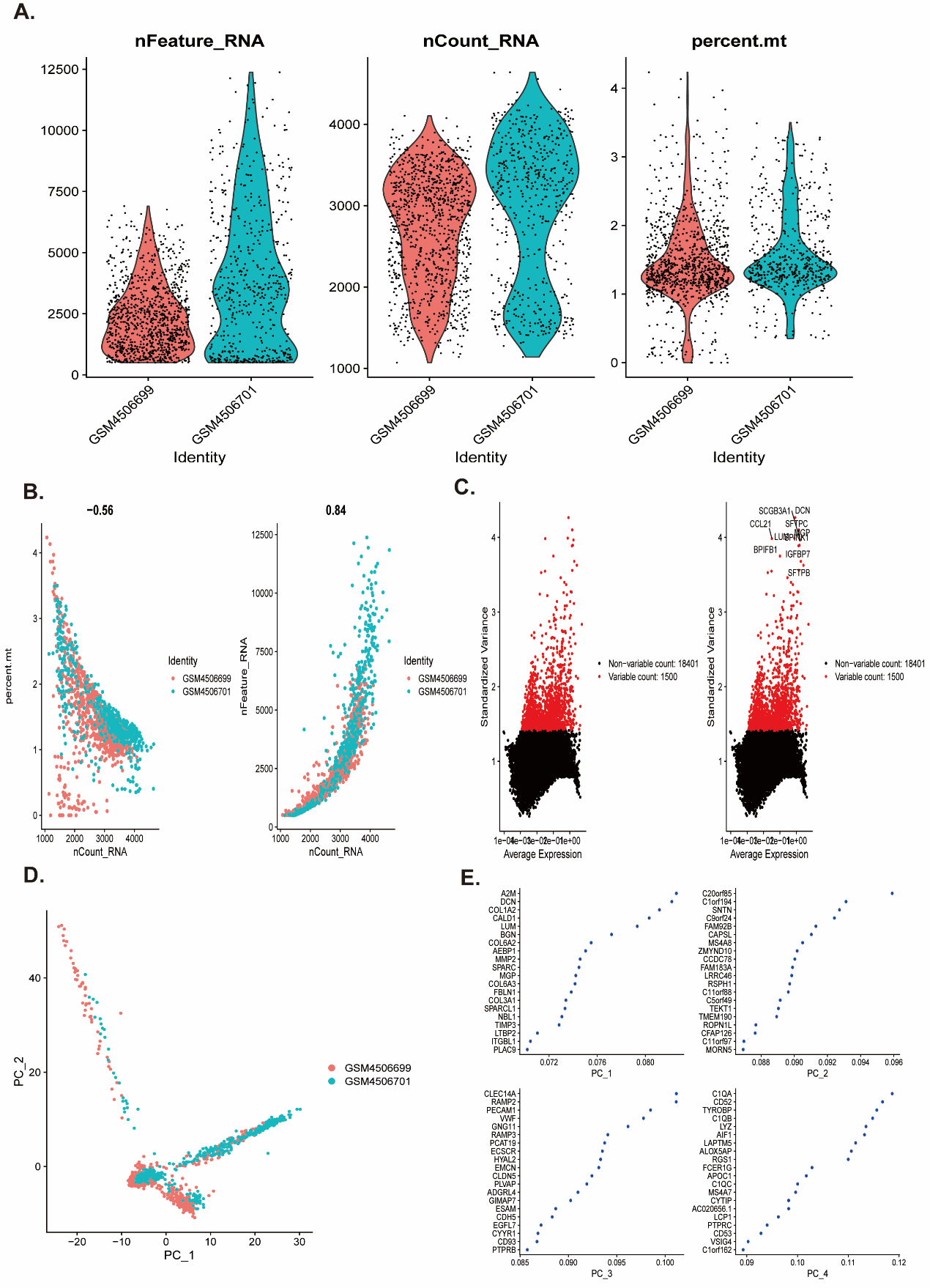
 **Figure S11. The quality control filtering of each sequenced cell from single cell sequening.** (A). Violin plots displayed their number of RNA features (nFeature_RNA) and absolute UMI counts (nCount_RNA) of each sample. (B). Correlation analysis between nFeature and nCount. (C). The top 1500 variable features were labeled in red. (D). PCA plot indicated that no significant differences in two samples. (D). The significant marked genes in four PC.


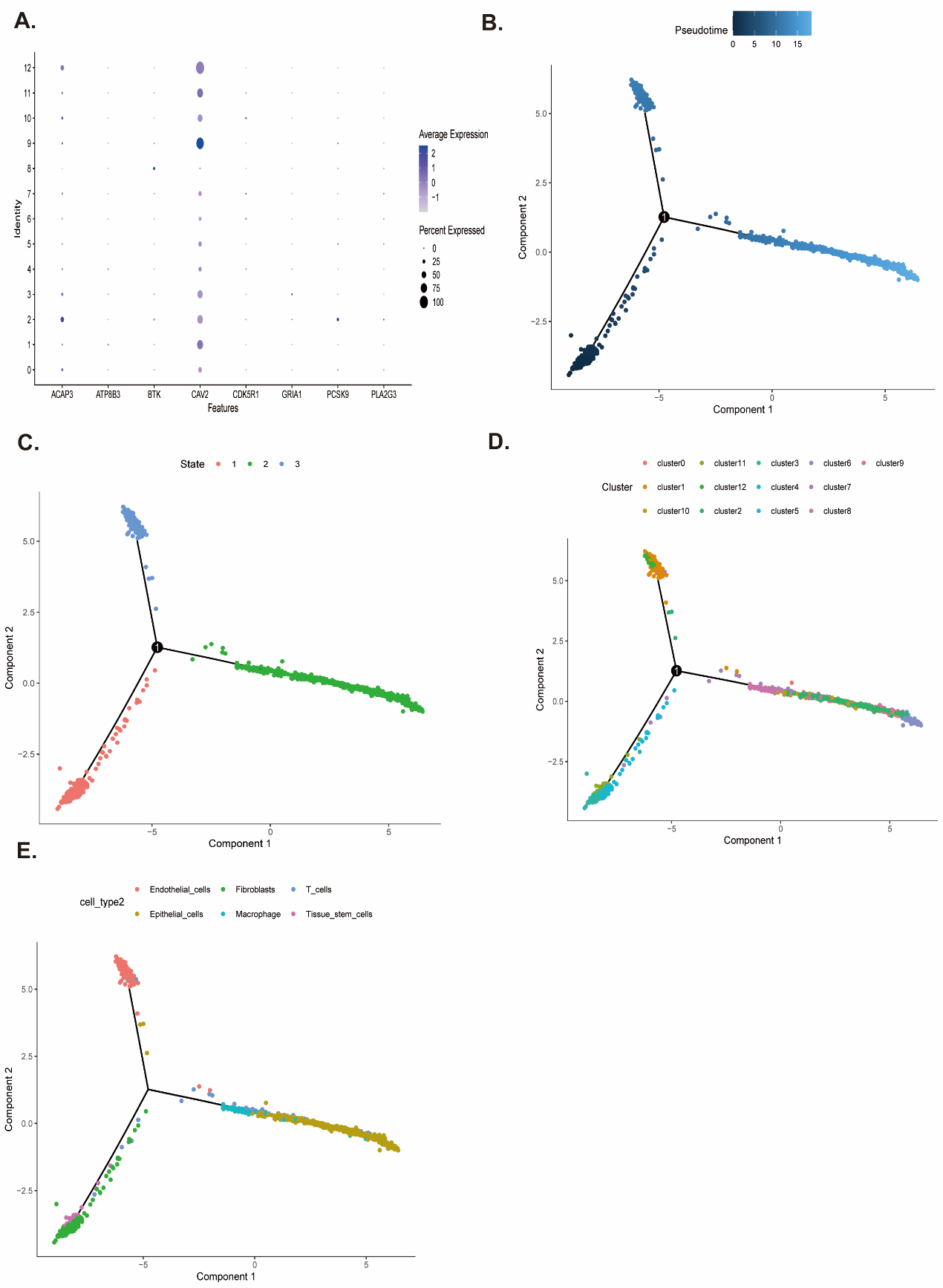
 **Figure S12. The pseudo-time trajectories of tumor and immune cells in LUAD.** (A). Dot plot of the eight prognostic genes expression of clusters. (B-E). Developmental trajectories of tumor cells ordered by pseudotime(B), and states(C), and displayed by clusters (D), and presented by cell types (E).
